# Minimum Time Search in Uncertain Dynamic Domains with Complex Sensorial Platforms

**DOI:** 10.3390/s140814131

**Published:** 2014-08-04

**Authors:** Pablo Lanillos, Eva Besada-Portas, Jose Antonio Lopez-Orozco, Jesus Manuel de la Cruz

**Affiliations:** 1 Mobile Robotics Lab, Institute of Systems and Robotics, University of Coimbra, Pinhal de Marrocos, Plo II, 3030-290 Coimbra, Portugal; E-Mail: planillos@isr.uc.pt; 2 Departamento de Arquitectura de Computadores y Automatica, Universidad Complutense de Madrid, Av. Complutense s/n, 28040 Madrid, Spain; E-Mails: jalo@dacya.ucm.es (J.A.L.-O.); jmcruz@fis.ucm.es (J.M.C.)

**Keywords:** multi-agent systems, Bayesian search theory, Minimum Time Search, Cross Entropy Optimization

## Abstract

The minimum time search in uncertain domains is a searching task, which appears in real world problems such as natural disasters and sea rescue operations, where a target has to be found, as soon as possible, by a set of sensor-equipped searchers. The automation of this task, where the time to detect the target is critical, can be achieved by new probabilistic techniques that directly minimize the Expected Time (ET) to detect a dynamic target using the observation probability models and actual observations collected by the sensors on board the searchers. The selected technique, described in algorithmic form in this paper for completeness, has only been previously partially tested with an ideal binary detection model, in spite of being designed to deal with complex non-linear/non-differential sensorial models. This paper covers the gap, testing its performance and applicability over different searching tasks with searchers equipped with different complex sensors. The sensorial models under test vary from stepped detection probabilities to continuous/discontinuous differentiable/non-differentiable detection probabilities dependent on distance, orientation, and structured maps. The analysis of the simulated results of several static and dynamic scenarios performed in this paper validates the applicability of the technique with different types of sensor models.

## 1. Introduction

According to the Theory of Optimal Search, a pioneering book by Lawrence D. Stone [1], “Search theory is one of the oldest areas of operation research”. In fact, the initial developments were already made by researches of the Operation Research Group of the US Navy during World War II. Subsequently the theory was used by the Navy for searching the H-bomb lost in 1966 in the Mediterranean Sea near Palomares, the submarine Scorpion lost in 1968 in the Atlantic Ocean near Azores and many other objects. Since then, the theory has been used for search and rescue as well as for many other nonmilitary applications [2]. Nowadays, much of the research aims at providing autonomous robots with the capability of searching and tracking.

The existing theory, which coexists in multiple research areas such as artificial intelligence, robotics, economics and statistics, has been developed under different assumptions related with the elements that appear in the different searching tasks. The usual common elements are the following: first, the target (or targets) under search; second, the agent (or agents) that perform the search using its moving capabilities to explore the space where the target stays; third, the sensor (or sensors), placed in the agents, that provide information about the searching space; forth, the environment that modifies the properties of the sensors and target interactions; fifth, the information management system that stores and updates the knowledge about the targets, agents and environment state; sixth, the searching strategy that controls the agents movements according to the available information. An finally, an additional element to consider is the uncertainty associated, not only to the target location, but also to the target displacements and to the sensorial information.

The interdisciplinary nature of the search problem is observed in the three main approaches used to formalize it mathematically. The first formulation, related to the operational research field and mainly used nowadays by the artificial intelligence and machine learning community, consists on considering it a Partially Observable Markov Decision Process (POMDP [3,4]) where the state of the target is unknown and partially/indirectly observed by the sensors. The second approach, coming from the control field, consists on considering it a stochastic optimal control problem with measurements uncertainty ([5,6]) where the controller outputs regulate both the dynamics of the system and the information gained by sensing. Finally, the third formulation, appearing in the data fusion community, exploits Bayes theory to (1) represent and update the knowledge of the problem state in the information management system; and to (2) formulate the searching strategy as an optimization problem with probabilistic information-based utility functions [7,8].

In a generic search problem, the information of the target state is modeled with a probability mass/density function over discrete/continous search spaces, and updated using appropriated Recursive Bayesian Filters (RBF [9,10]), such as the RBF for probability masses used in [11–13] or the extended Kalman and Particle Filters used in [7,14,15]. The utility function to be optimized is not always the same either, and varies from the system entropy used in [11] to the logarithmic information gain in [16], the probability of detecting (or no-detecting) the target during the decision plan in [8,17–24], the expected detection time in [12,13,25], the expected number of detections in [26], the expected estimation covariance associated to the sensor observations in [7,14], the expected log-likelihood of the posterior target distribution associated to the sensor observations in [15], or the intensity/strength of the observation in source seeking problems [27–29]. The high complexity of the different variants of the problem is also tackled with a big variety of techniques, ranging from POMDP solvers [17] to branch and bound approaches [18–20,23,26], genetic algorithms [19], greedy search [8,16,21], gradient descendent methods [22,24], limited depth first search [25], Cross Entropy Optimization [12] and bayesian optimization algorithms [13]. Finally, it is worth noting that new versions of the problem and solutions are continuously appearing, driven by the interest of the search in multiple real-world applications, the development of small and cheap unmanned vehicles/sensors, and the inherent beauty and complexity of the problem.

This paper is focused in the analysis of the applicability of one of the existing utility functions to critical time search problems where real-world sensors, with complex sensorial models, are being used. The interest on these types of problems, where the target under search has to be found as soon as possible, appears in many search applications such as natural disasters or sea rescue operations. The development of the solution under test in this paper, partially presented by the authors in [12,13], was motivated by the reduced number of generic multi-agent searching algorithms that are capable of (1) dealing with different types of real-world sensors and target dynamics; and of (2) optimizing a probabilistic utility function directly related with the search time. The proposed solution consisted on exploiting the detection capabilities of the sensors to implement a Minimum Time Search (MTS) strategy that minimizes the Expected Time (ET) to detect the target using Cross Entropy Optimization (CEO [30]), which is a probabilistic optimization algorithm with global searching capabilities for combinatorial problems with complex non-linear/non-differential utility functions.

The promising properties of the ET-CEO based solution have only been tested in [12] with ideal sensors with a binary (0/1) probability of detection model a streamlined sensorial model (where the probability of detection is zero whenever the agent does not observe the target, and one otherwise) that appears also in other formulations of the search problem [17,25]. In fact, simplified sensorial models are commonly used in other solutions of the search problem that are closely related to the ET utility function, because they simplify the problem formulation and/or are required by the selected optimization method. For example, stepped detection probability functions with zero/constant probability outside/inside the observable region are used in the entropy-based utility function presented in [11], and continuous differentiable distance-based detection probability functions are employed in the joint detection-probability function used in [8,21,22,24]. Therefore, the evaluation presented in this paper of the ET-CEO-based method with several complex models will provide additional information of (1) the applicability of the method to complex sensorial models and of (2) the possibility of using CEO as the optimizer of other related utility functions without simplifying the sensorial models of the system.

This paper also complements the outlined presentation of the multi-agent MTS problem [13] and ET-CEO solution [12] with a *detailed* mathematical definition of the problem, an algorithmic presentation of the different elements of the solution, and a mathematical derivation of the expressions used within the MTS system. Additionally, it includes the mathematical formulation of different minimum time search problems, paying a special attention to the description of their sensorial models.

This paper is organized as follows. Section 2 presents the mathematical formulation of the MTS problem and algorithmically details the generic ET-CEO approach used to solve it. Section 3 collects the most relevant characteristics of existing closely related publications and states the differences that exist among them and this work. Section 4 describes the experimental setups that are analyzed in this paper, which make use of three different complex sensorial models associated to a radar working in a space free of obstacles, an ultrasonic scanner working in a structured environment, and a camera and vision identification system working in orientation-dependent scenarios. Section 5 shows and analyzes the results obtained under simulation by the MTS solution presented in Section 2 in static and dynamic scenarios of the experiment setups described in Section 4. Finally, the conclusions of the work are drawn in Section 6 and the Appendix collects the mathematical derivations of the equations used to tackle the problem.

## 2. Minimum Time Search

The minimum time search problem in uncertain dynamic domains tackled in this paper is a searching task that involves two types of dynamic partakers: the target (searched element) and the agents (searchers). The target is an object/person that moves freely over a limited searching region, starting in an unknown location (state). The agents are the vehicles/robots that following the control actions obtained by a searching strategy move themselves over the searching region with the purpose of detecting, using their sensors, the target. In the MTS problem, the purpose of the searching strategy is to determine the best set of actions that will let the agents find (detect) the target as soon as possible, taking into account the uncertainty associated to the target state, target displacements and sensorial information.

To tackle the problem, we complete the problem definition with probability models associated to the problem uncertainty sources and use Bayes inference as the core of the information gathering system and of the searching strategy. Our solution to the MTS problem is a perception-action loop [31] with two interacting layers. The first, called data fusion layer hereafter, is the information gathering system that updates the probability of locating the target in any of its possible states using the detection measurements provided from the agents current location. The second, called controller layer hereafter, is the searcher strategy that obtains the sequence of actions that will make the agents move and collect observations from the positions (states) that will minimize the expected time of target detection.

The rest of the section is divided in two parts. In the first, we present a formal definition of all the elements that constitute the MTS problem. In the second, we describe the algorithms implemented within the data fusion and controller layer of the ET-CEO searching strategy under test by several complex sensorial models.

### 2.1. Problem Definition

In this section we introduce the variables used to define the properties of the elements of the MTS problem, as well as the probability functions employed to model the uncertainty sources, and the underlying assumptions performed during the problem formulation:

- Ω represents the space where the target is contained and where the search is done. We assume that Ω is discrete and defined by the finite number of cells of a multi-dimensional grid;
- *τ^k^* ∈ Ω is the random variable that represents the unknown target state at time step *k*;
- *M* is the number of agents that take active part in the search making observations of the environment;
- Δ stands for the space of possible actions that can be applied to each agent. We assume that Δ is discrete and defined by a finite number of possibilities;
- $u_{i}^{k}\in \Delta$ is the control action that the i-th agente should apply at time step *k*;
- $s_{i}^{k}$ the known state of the *i*-th agent at time step *k*. Its value is completely determined by the previous agent location $s_{i}^{k-1}$ and the current control action $u_{i}^{k}$. In other words, given an initial agent location $s_{i}^{k}$ and a sequence of *N* control actions $u_{i}^{k+1:k+N}=\left\{u_{i}^{k+1},u_{i}^{k+2},\cdots ,u_{i}^{k+N}\right\}$, the deterministic trajectory of the agent $s_{i}^{k:k+N}=\left\{s_{i}^{k},s_{i}^{k+1},s_{i}^{k+2},\cdots ,s_{i}^{k+N}\right\}$, including the starting state $s_{i}^{k}$, is completely defined and viceversa;
- $z_{i}^{k}$ is the measurement taken by the sensor placed at agent *i* at time step *k*. In the discrete space MTS problem, we only consider two possible observations: detection *D* and no detection *D̅*. To simplify the expressions, we will also represent $z_{i}^{k}=D$ as $D_{i}^{k}$ and $z_{i}^{k}=\bar{D}$ as ${\bar{D}}_{i}^{k}$
- *ϵ* represents the known state of the environment. It is used to define the environmental and fixed parameters that modify the behavior of the target and the sensors;
- *b*(*τ*^0^) = *P*(*τ*^0^) stands for the initial target state belief. That is, *b*(*τ*^0^) is used to model the initial state uncertainty as the probability *P*(*τ*^0^). Additionally, $b(\tau^{k})=P\left(\tau^{k}|z_{1:M}^{1:k},s_{1:M}^{0:k},\epsilon \right)$ is the belief or probability of the target state *τ^k^* at time step *k* given the environment state ϵ, and all the measurements $z_{1:M}^{1:k}$ and agent trajectories $s_{1:M}^{0:k}$ up to *k*;
- *P*(*τ^k^*|*τ^k^*^−1^,*ϵ*) represents the target transition model or the probability of the target moving from state *τ^k^*^−1^ to state *τ^k^* given the environment state. As we assume that the target is not evading from the agents, the selected model shows no dependency of the target state *τ^k^* on the agents states $s_{1:M}^{k-1}$;
- $P\left(z_{i}^{k}=D|\tau^{k},s_{i}^{k},\epsilon \right)$ is the sensor likelihood or probability of detecting the target given the target *τ^k^*, agent $s_{i}^{k}$ and environment *ϵ* states. The probability of not detecting the target is defined as $P(z_{i}^{k}=\bar{D}|\tau^{k},s_{i}^{k},\epsilon )$ and is complementary to the sensor likelihood, *i.e.*, $P\left(z_{i}^{k}=D|\tau^{k},s_{i}^{k},\epsilon \right)=1-P\left(z_{i}^{k}=\bar{D}|\tau^{k},s_{i}^{k},\epsilon \right)$. As we assume that the measurements of each agent are independent on the information of other agents, the sensor model shows no dependency of $z_{i}^{k}$ on any other (*j* ∈ {1 : *M*} − {*i*}) agent state $s_{j}^{k}$ or measurement $z_{j}^{k}$.

The objective in the MTS problem is to determine the actions that will drive the agents to find the target as soon as possible. Formally, the objective of the MTS is to determine the joint sequence of N actions $u_{1:M}^{k+1:k+N}=\left\{u_{1}^{k+1:k+N},u_{2}^{k+1:k+N},\cdots ,u_{M}^{k+1:k+N}\right\}$ that minimizes the time of finding the target, given the environment state ϵ, agents states $s_{1:M}^{k}$, target state belief $b_{\tau }^{k}$, target transition model *P*(*τ^k^*|*τ^k^*^−1^, *ϵ*) and agents' measurement model $P\left(z_{i}^{k}|\tau^{k},s_{i}^{k},\epsilon \right)$

### 2.2. Problem Solution

**Figure 1.**
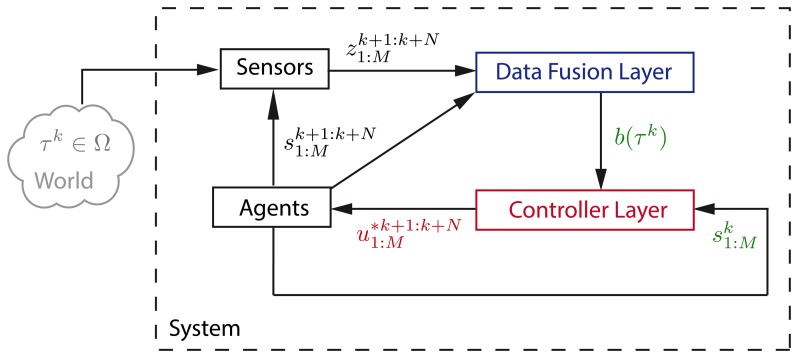
General system structure.

Our solution to the MTS problem is the autonomous and intelligent system abstractly represented in Figure 1 as a perception-action loop [31]. Its general processing structure, similar to the one in [8,11,32], consists in two interacting layers: (1) the data fusion layer that is in charge of calculating the target state belief *b*(*τ^k^*) with the measurements $z_{1:M}^{1:k}$ provided by the agents' sensors; and (2) the controller layer that has to compute the optimal joint sequence of actions $u_{1:M}^{k+1:k+N}$ using the data fusion layer's target state belief *b*(*τ^k^*), the agents location $s_{1:M}^{k}$, and the target transition and observation models, *i.e.*, *P*(*τ^k^*|*τ^k^*^−1^, *ϵ*) and $P\left(z_{i}^{k}|\tau^{k},s_{i}^{k},\epsilon \right)$

In the following, we detail the operations performed in each layer and their interactions, showing the cyclic relationship that occurs within the perception-action loop. We also provide the algorithms that compute those operations. Their complete derivation can be found in the Appendix.

#### 2.2.1. Data Fusion Layer

This processing layer is implemented as a Recursive Bayesian Filter (RBF [9,10]) that calculates the target state probability or belief *b*(*τ^k^*) at time step *k* using the real measurements $z_{1:M}^{k}$ provided by the agents' sensors, the previous time step belief *b*(*τ^k^*^−1^), the target transition model and the measurement model. The process starts from the given initial target belief *b*(*τ*^0^) and obtains *b*(*τ^k^*) iterating the following two steps:

1. The *prediction* step calculates with Equation (1) the probability $P\left(\tau^{k}|z_{1:M}^{1:k-1},s_{1:M}^{0:k},\epsilon \right)$ of the target state at time step *k* given the previous target belief *b*(*τ^k^*^−1^) and the target transition model. In other words, it estimates the target location at time step *k* considering the previous target belief that only takes into account the previous measurements $z_{1:M}^{1:k-1}$
  (1) $$P\left(\tau^{k}|z_{1:M}^{1:k-1},s_{1:M}^{0:k},\epsilon \right)=\sum_{\tau_{k-1}\in \Omega }P\left(\tau^{k}|\tau^{k-1},\epsilon \right)b\left(\tau^{k-1}\right)$$
2. The *update* step calculates with Equation (2) the target belief $b(\tau^{k})=P\left(\tau^{k}|z_{1:M}^{1:k},s_{1:M}^{0:k},\epsilon \right)$ at time step *k* using the sensorial information $z_{1:M}^{k}$ of that time step, the measurement model, and the probability $P\left(\tau^{k}|z_{1:M}^{1:k-1},s_{1:M}^{0:k},\epsilon \right)$ obtained in the prediction step. In other words, it includes in the target belief at time step *k* the information of the previous measurements $z_{1:M}^{1:k-1}$ and of the measurements $z_{1:M}^{k}$ taken at time step *k*.
  (2) $$\begin{array}{c} b(\tau^{k})=\eta^{-1}\underset{i=1:M}{\text{∏}}P(z_{i}^{k}|\tau^{k},s_{i}^{k},\epsilon )P(\tau^{k}|z_{1:M}^{1:k-1},s_{1:M}^{0:k},\epsilon )\\ \text{with}\;\eta =\underset{\tau_{k}\in \Omega }{\text{∑}}\underset{i=1:M}{\text{∏}}P(z_{i}^{k}|\tau^{k},s_{i}^{k},\epsilon )P(\tau^{k}|z_{1:M}^{1:k-1},s_{1:M}^{0:k},\epsilon ) \end{array}$$

In short, the prediction step modifies the target state probability distribution taking into account the target transition model while the update step modifies its value according to the new set of measurements. The information and operations used to perform both steps are presented in Algorithm 1, where the update step has been divided in three operations and *b̂*(*τ^k^*) represents the target belief after the transition and measurement inclusion, before being normalized.



**Algorithm 1** Bayesian Recursive Filter
**Require**: *b*(*τ^k^*^−1^)▹ Prior target state belief**Require**: 
$s_{1:M}^{k}$▹ Current agents state**Require**: 
$z_{1:M}^{k}$▹ Current measurements**Require**:*P*(*τ^k^*|*τ^k^*^−1^, *ϵ*)▹ Target transition model**Require**: 
$P(z_{i}^{k}|\tau^{k},s_{i}^{k},\epsilon )$▹ Observation model1: 
$P(\tau^{k}|z_{1:M}^{1:k},s_{1:M}^{0:k},\epsilon )=\sum_{\tau_{k-1}\in \Omega }P(\tau^{k}|\tau^{k-1},\epsilon )b(\tau^{k-1})$▹ Prediction step2: 
$\hat{b}(\tau^{k})=\prod_{i=1:M}P(z_{i}^{k}|\tau^{k},s_{i}^{k},\epsilon )P(\tau^{k}|z_{1:M}^{1:k-1},s_{1:M}^{0:k},\epsilon )$▹ Udpate step: unnormalized belief3: 
$\eta =\sum_{\tau^{k}\in \Omega }\hat{b}\left(\tau^{k}\right)$▹ Udpate step: normalization factor4: 
$\hat{b}\left(\tau^{k}\right)=\eta^{-1}\hat{b}\left(\tau^{k}\right)$▹ Udpate step: normalized belief5: **return**
*b*(*τ^k^*)


#### 2.2.2. Controller Layer

In the MTS process, the controller layer has to compute the joint sequence of actions $u_{1:M}^{k+1:k+N}$ that optimizes the searching time. In order to do that, we optimize a *utility function* directly related with the search time using an approximated *optimization algorithm* capable of handling the inherent complexity of the search task. The selected utility function and optimization technique are the following:

**The utility function** to be minimized is the *Expected Time* (ET) to detect the target for a limited horizon of N time steps [13]. As Equation (3) states, the ET function accumulates the probability of no detecting the target from the proposed following N agents states $s_{1:M}^{k+1:K+N}$. To calculate it, we use Equations (4)–(6), where *f*(*τ^k^*^+1^) represents the prediction of the belief *b*(*τ^k^*), *f*(*τ^k^*^+^*^j^*) is obtained recursively including in *f*(*τ^k^*^+^*^j^*^−1^) the no-detection measurements with an unnormalized update step and predicting the obtained value to the *k* + *j* time step, and ET ( $s_{1:M}^{k:k+N}$) accumulates, for each time step within the horizon, the values obtained by including the no-detection measurements in *f*(*τ^k^*^+^*^j^*).
  The information and expressions used to calculate ET for the agents trajectories $s_{1:M}^{k+1:k+N}$ and target belief *b*(*τ^k^*) are presented in Algorithm 2, where the operations have been reorganized to perform the measurements updates in Equations (4) and (5) only once and *b̂*(*τ^k^*^+^*^j^*) represents the remaining probability, obtained after predicting and including the no-detection measurements without the normalization operation of the update step of the RBF. It is important to highlight that the computation cost of the ET evaluation, as Algorithm 2 states, is proporcional to the number of actions *N* in the “for” loop, the number of sensor updates in the product $\Pi_{i=1}^{M}$ and number of states in the Σ*_τ^k^_*_^+^_*_^j^_*, *b*(*τ^k^*^+^*^j^*) and *f*(*τ^k^*^+^*^j^*). Besides, the ET utility function can be defined over the agents trajectories $s_{1:M}^{k+1:k+N}$ instead of over the joint sequence of control actions $u_{1:M}^{k+1:k+N}$ due to the deterministic relationship that exists between { $s_{1:M}^{k}$, $u_{1:M}^{k+1:k+N}$ } and $s_{1:M}^{k+1:k+N}$.
  (3) $$\text{ET}(s_{1:M}^{k+1:k+N})=\sum_{j=1}^{N}P({\bar{D}}_{1:M}^{k+1:k+j}|s_{1:M}^{1:k+j},z_{1:M}^{1:k},\epsilon )=$$
  (4) $$=\sum_{j=1}^{N}\sum_{\tau^{k+j}\in \Omega }\prod_{i=1}^{M}P({\bar{D}}_{i}^{k+j}|\tau^{k+j},s_{i}^{k+j},\epsilon )f(\tau^{k+j})$$
  (5) $$\text{with}\;f(\tau^{k+j})=\sum_{\tau^{k+j-1}\in \Omega }P(\tau^{k+j}|\tau^{k+j-1})\prod_{i=1}^{M}P({\bar{D}}_{i}^{k+j-1}|\tau^{k+j-1},s_{i}^{k+j-1})f(\tau^{k+j-1})$$
  (6) $$f(\tau^{k+1})=\sum_{\tau^{k}\in \Omega }P(\tau^{k+1}|\tau^{k},\epsilon )b(\tau^{k})$$
**Algorithm 2** Expected Time Utility Function
**Require:**
*b*(*τ^k^*)▹ Current target state belief**Require:**
$s_{1:M}^{k+1:k+N}$▹ Proposed agents state trajectory**Require:**
*P*(*τ^k^*|*τ^k^*^−1^,ϵ)▹ Target transition model**Require:**
$P(z_{i}^{k}=\bar{D}|\tau^{k},s_{i}^{k},\epsilon )$▹ Non detection observation model1: *ET* ← 0▹ Initialize ET variable2: 
$f(\tau^{k+1})\leftarrow \sum_{\tau^{k}\in \Omega }P(\tau^{k+1}|\tau^{k},\epsilon )b_{\tau }^{k}$▹ Transition to next time step3: **for** j=1:N **do**4:  
$\hat{b}(\tau^{k+j})\leftarrow \Pi_{i=1}^{M}P({\bar{D}}_{i}^{k+j}\left|\tau^{k+j},s_{i}^{k+j})f(\tau^{k+j}\right)$▹ Unnormalized ‘belief’5:  *ET*←*ET* + ∑*_τ^k^_*_^+*j*^_
*b̂*(*τ^k^*^+^*^j^*)▹ Update ET accumulating the unnormalized ‘belief’6:  
$f\left(\tau^{k+j+1}\right)\leftarrow \sum_{\tau^{k+j}\in \Omega }P\left(\tau^{k+j+1}|\tau^{k+j},\in \right)\hat{b}\left(\tau^{k+j}\right)$▹ Transition to next time step7: **end for**8: **return**
*ET*
**The optimization algorithm** used to minimize ET is Cross Entropy Optimization (CEO [30]), a method capable of tackling successfully complex optimization combinatorial problems. In our MTS problem, CEO looks for the optimal solution (actions sequence, *i.e.*, $u_{1:M}^{k+1:k+N}$) of the utility function (ET, Algorithm 2). It alternates the estimation of the action sequence probability distribution *q^l^*^+1^ from the subset of best solutions *U'^l^* obtained by the algorithm in the previous iteration with the creation of a new set of solutions sampled accordingly to the existing action sequence probability distribution *q^l^*. This way of proceeding helps CEO to avoid local minima and sequentially approximates the action sequence probability distribution *q^l^* to the probability distribution of the *best* action sequence q*. Additionally, CEO assumes that the variables within each solution (*i.e.*, the actions to be performed by each agent in each of the N steps of the sequence) are independent on each other. This has three important consequences. On one hand, it implies that the action sequence probability distribution *q^l^* can be defined as a |Δ| · *N* probability table where each column represents one of the action variables $u_{i}^{k+j}$ within $u_{1:M}^{k+1:k+N}$ and each row is associated to the probability of each of the actions in Δ for the corresponding action variable $u_{i}^{k+j}$ *.* On the other hand, the value of $u_{i}^{k+j}$ can be directly sampled from the probability distribution stored in its associated column in *q^l^*. Finally, *q^l^*^+1^ can be efficiently estimated from a subset of solutions counting the number of times that each variable $u_{i}^{k+j}$ takes, within the subset of solutions, each of possible values of the action space Δ.
  The steps of the implementation of CEO for the MTS problem are presented in Algorithm 3. In the first step, it generates the actions sequence probability distribution *q*^0^ assuming that all the actions are equally probable for all the agents and steps of the sequence. That is, it makes all the entries in the probability table *q*^0^ equal to 1/|Δ|. The steps within the “for” loop, used to create and evaluate the new set of possible solutions $U_{1:E}^{l}$, sample each possible solution (actions sequence, $U_{e}^{l}≜u_{1:M}^{k+1:k+N}$) from the actions sequence probability table *q^l^*; obtain the agents trajectories $S_{e}^{l}≜s_{1:M}^{k+1:k+N}$ associated to the initial agents locations $s_{1:M}^{k}$ and sampled action sequence $U_{e}^{l}$; and calculate, using Algorithm 2, the ET to detect the target $J_{e}^{l}$ given the belief *b*(*τ_k_*), and transition and measurement models P(*τ^k^*|*τ^k^*^−1^, *ϵ*) and $P(z_{i}^{k}|\tau^{k},s_{i}^{k},\epsilon )$. Afterwards, CEO sorts the solutions according to their ET and selects the subset of solutions (actions sequences) whose ET is smaller than the ET of the solution which is placed in the *round*(*ϰE*) position of the sorted list of solutions. That is, it selects a small percentage of solutions whose value is smaller than the *round*(*ϰE*)-th best value of the existing solutions $U_{e}^{l}$. In the following two steps, the new actions sequence probability table *q^l^*^+1^ is obtained (1) using the information in the subset of best solutions and (2) smoothing the values of the new actions sequence probability table *q^l^*^+1^ with the values of the previous actions sequence probability table *q^l^*. The process continues until a fixed number of iterations is reached or the changes of the values in the probability table are negligible. At the end, the algorithm returns the best solution $u_{1:M}^{*k+1:k+N}$ sampled by the algorithm during all their iterations. Note that this solution is not necessary the optimum of the ET function, only the best found by CEO.

**Algorithm 3** Cross Entropy Optimization Algorithm
**Require:**
*b*(*τ^k^*)▹ Prior target location belief**Require:**
$s_{1:M}^{k}$▹ Initial agents location**Require:**
*P*(*τ^k^*|*τ^k^*^−1^,*ϵ*)▹ Target transition model**Require:**
$P(z_{i}^{k}|\tau^{k},s_{i}^{k},\epsilon )$▹ Observation model**Require:**
*E*▹ Number of samples**Require:**
*ϰ*▹ Percentil of best solutions**Require:**
*α*▹ Smoothing factor1: *q*^0^ ← Initialize uniformly the actions sequence distribution▹ *Initialize q*^0^2: *l* ← 0▹ Set iteration index3: **while** no finished **do**▹ Iteration loop4:   **for**
*e* = 1 to *E*
**do**▹ *Create and evaluate new set of solutions*5:    
$U_{e}^{l}\leftarrow u_{1:M}^{k+1:k+N}∼q^{l}$▹ Sample new actions sequences6:    
$S_{e}^{l}\leftarrow s_{1:M}^{k+1:k+N}\leftarrow \left\{s_{1:M}^{k},U_{e}^{l}\right\}$▹ Translate actions sequences to trajectories7:    
$J_{e}^{l}\leftarrow \text{ET}\left(b\left(\tau^{k}\right),S_{e}^{l},P\left(\tau^{k}|\tau^{k-1},\epsilon \right),P\left(z_{i}^{k}=\bar{D}|\tau^{k},s_{i}^{k},\epsilon \right)\right)$▹ Evaluate trajectories with Alg. 28:   **end for**9:   
$\left[U_{1:E}^{sort,l},J_{1:E}^{sort,l}\right]=sort(U_{1:E}^{l},J_{1:E}^{l})$▹ Sort solutions (lower first) according to 
$J_{1:E}^{l}$10:   
$U{'}^{l}\leftarrow \left\{U_{e}^{l}|U_{e}^{l}\in U_{1;E}^{l}\wedge J_{e}^{l}\leq J_{round(\varkappa E)}^{sort,l}\right\}$▹ *Select best subset of solutions*11:   *q^l^*^+1^ ← Learn new action list distribution from *U'^l^*▹ *Estimate q^l^*^+1^12:   *q^l^*^+1^ ← *αq^l^*^+1^ + (1 − *α*)*q^l^**>* Smooth *q^l^*^+1^ distribution with *q^l^*13:  *l* ← *l* + 114: **end while**15: **return**
$u_{1:M}^{*k+1:k+N}$▹ Solution with best 
$J_{1:E}^{1:l-1}$

There are several reasons to select this utility function and optimization algorithm. On one hand, in critical time searching tasks, the sooner the target is detected, the higher the chances to find it sooner. Therefore, in these types of tasks it is beneficial to optimize a utility function directly related with the detection time, such as it is expected value (*i.e.*, ET). On the other hand, the inherent complexity of the searching task, at least NP-hard [33], should be tackled with a computational efficient optimization method that is able to deal with the multiple local minima that can appear in the landscape of the ET utility function due to the target belief *b*(*τ_k_*), target transition *P*(*τ^k^*|*τ^k^*^−1^, *ϵ*) and no detection likelihood $P({\bar{D}}_{i}^{k}|\tau^{k},s_{i}^{k},\epsilon )$. CEO is a good choice in this respect, as its probabilistic nature and global searching capabilities allow it to navigate through complex non-linear/non-differential utility function landscapes, and its computation cost can be controlled by fixing the maximum iterations and number of samples. Other possible choices of utility function and heuristic for the controller layer are discussed in Section 3.

#### 2.2.3. Interaction and Data Flow between the Data Fusion and Controller Layers

The cyclic interactions between the different parts of the system of the MTS problem, which are schematized in Figure 1, are the following:

(1) At time step *k*, the controller layer uses Algorithm 3 to determine the sequence of the following N agents actions $u_{1:M}^{*k+1:k+N}$, based on the current agents states $s_{1:M}^{k}$, the target transition P(*τ^k^*|*τ^k^*^−1^, *ϵ*), the no-detection likelihood $P({\bar{D}}_{i}^{k}|\tau^{k},s_{i}^{k},\epsilon )$ and the belief *b*(*τ^k^*) obtained by the data fusion layer.
(2) Next, the agents execute the actions *sequentially* while the data fusion layer updates the belief. The way of proceeding at time step *k* + *j* (with *j* ∈ {1 : *N*}) consists in:
  (a) Applying actions $u_{1:M}^{*k+j}$ to displace the agents from $s_{1:M}^{k+j-1}$ to $s_{1:M}^{k+j}$;
  (b) Collecting, when the agents are placed at the states $s_{1:M}^{k+j}$, a new observation set $z_{1:M}^{k+j}$ related with the unknown target state *τ^k^*^+^*^j^*;
  (c) Making the data fusion layer update the belief *b*(*τ^k^*^+^*^j^*) from the belief *b*(*τ^k^*^+^*^j^*^−1^) using Algorithm 1, the current agents location $s_{1:M}^{k+j}$ and the collected set of observations $z_{1:M}^{k+j}$;
  (d) Incrementing j (*i.e.*, *j* = *j* + 1);
  (e) Returning to step (a) if *j* < *N* + 1 and all the observations in $z_{1:M}^{k+j}$ correspond to no-detection events. Otherwise, the current updated belief *b*(*τ^k^*^+^*^j^*) is sent to the controller layer to restart the cyclic iteration at step (1) from time step *k* + *j* − 1.

This way of proceeding makes the controller layer obtain the action sequence $u_{1:M}^{*k+1:k+N}$ after at most *N* iterations of the data fusion layer. In order to speed up the controller operations, we can re-start the CEO Algorithm to calculate the next sequence of actions $u_{1:M}^{*k+N+1:k+2N}$ as soon as it has returned the current one $u_{1:M}^{*k+1:k+N}$, by performing the *N* iterations of the data fusion layer assuming that the next *N* sets of measurements will be no-detection. If that is the case, *i.e.*, there has been only no-detection measurements during the execution of the sequence of actions, the controller will have the next sequence of actions available when the agents have performed the previous action sequence. If it is not, *i.e.*, there is a detection measurement available at time step *k* + *j*, the data fusion layer will have to update the belief *b*(*τ^k^*^+^*^j^*) with the real measurements, and the controller layer will have to be re-started to obtain the action sequence $u_{1:M}^{*k+j+1:k+j+N}.$

## 3. Related Work

In this section we discuss multiple works related with the formulation and solution presented in this article to tackle the MTS problem. We approach the discussion stating the relationship of the MTS problem with Partially Observable Markov Decision Processes (POMDPs [3,4]) and presenting some of the solutions used to tackle it under this perspective. Alternative solutions, closely related to ours are presented later and compared against each other based on several properties, including the observation models used to test their performances.

If we consider the elements and probability models of the MTS problem definition in Section 2.1, it seems natural to formulate it as POMDP, with a state defined by {*τ^k^*, $s_{1:M}^{k}$ }, an observation associated to $z_{1:M}^{k}$, an action represented by $u_{1:M}^{k}$, a transition probability model characterized by $P(\tau^{k}|\tau^{k-1},\epsilon )\cdot \prod_{1:M}P(s_{i}^{k}|s_{i}^{k-1},u_{i}^{k},\epsilon )$ where the transition agent probability function $P\left(s_{i}^{k}|s_{i}^{k-1},u_{i}^{k},\epsilon \right)$ is a delta function over the agent state $s_{i}^{k}$ reached deterministically from $s_{i}^{k-1}$ after applying $u_{i}^{k}$, an observation probability model calculated as $\prod_{1:M}P(z_{i}^{k}|\tau^{k},s_{i}^{k},\epsilon )$, and a reward function, $R\left(\left\{s_{1:M}^{k},\tau^{k}\right\},u_{1:M}^{k}\right)$, required in the POMDP formulation to define the function optimized by the controller, which is the expected accumulated reward $E\left[\overset{N}{\underset{j=1}{\text{∑}}}R([s_{1:M}^{k+1},\tau^{k+j}],u_{1:M}^{k+1})|b(\{\tau^{k},s_{1:M}^{k}\})\right]$ obtained by the agents given the initial system belief *b*({*τ^k^*, $s_{1:M}^{k}$}). However, formulating a problem under the POMDP perspective does not directly solve it, it is still necessary to select an appropriated method to tackle it.

The direct approach to solve a POMDP problem is to use a POMDP algorithm. In this last case, although *exact* POMDP techniques (capable of finding the optimal solution of POMDP problems with small spaces) are not valid due to the big state space of the MTS problem, *approximated* recently developed POMDP model based methods [34–36] and model-free algorithms [37], used successfully to solve other tracking [38–40] and detection/recognition [41] problems, can be applied. Although the model-free approaches, also used in other control problems [42,43], have the advantage of not requiring the knowledge of the probabilistic transition and sensorial models, the method used in this paper and the techniques analyzed hereafter, exploit these knowledge to tackle search problems.

Alternatively, it is possible to exploit the inner characteristics of the MTS problem, to reduce the complexity of its formulation as a POMDP, and then solve it with an adapted strategy. Some characteristics to be considered are (1) the deterministic behavior of the agents; (2) the binary detection/no-detection nature of the sensor model; and/or (3) the fact that in the definition of some of the search detection-based utility functions (such as the probability of not detecting the target during the decision plan and the expected time to detect it) only the no-detection measurement event appears naturally. Examples of this strategy are presented in [17] and [32]. In the first, [17], the author exploits the first two facts to maximize the probability of detecting the target during the decision plan by optimizing, using an adapted exact POMDP technique which is only valid for problems with small state spaces [44], a value-function defined over the target belief *b*(*τ^k^*) and a single-agent state $s_{1}^{k}$. In the second, [32], the author focuses on the third fact to take out the expectation over the possible measurements in the POMDP utility function associated to the probability of not detecting the target during the decision plan. He goes a step further, and instead of optimizing the problem with an adapted POMDP algorithm, he does it directly with a gradient-based method. In other words, although he defines the search problem as a POMDP, he finally uses a deterministic optimization method to tackle it.

Another option is to ignore the POMDP nature of the search, and formulate it as a bayesian inference problem [10,31] using appropriated utility functions and optimization algorithms. This is the strategy followed, for instantce, in [8,11–13,16,21,22,24,25] to tackle the search problem with different detection-based probability utility functions and types of algorithms. The used utility functions, which are capable of managing the prior information and uncertainty sources of the search problem, are: the system entropy [11], the logarithmic information gain [16], the probability of detecting (or no-detecting) the target during the decision plan [8,21,22,24], the expected detection time [12,13,25], and the discounted probability of detecting the target during the decision plan [12,13]. Among these functions, the best options for the MTS problem are the optimization of the expected time and of the discounted probability of detecting the target during the decision plan, because the first is directly related, by definition, with the detection time, and the second includes a discounting factor that rewards the detection of the target in the initial steps of the decision plan. Moreover, optimizing any of the other options does not optimize the time necessarily [12,25]. The used approximated optimization algorithms are diverse: greedy search in [8,16,21], gradient descent on [22,24], neural networks in [11], limited depth first search in [25], CEO in [12] and Bayesian Optimization Algorithm (BOA) in [13]. Other differentiable factors of these works are the definition of the action space Δ (discrete in [11–13,25] and continuous in [8,16,21,22,24]), the number of agents on the formulation (one in [12,25] and several in [8,11,13,16,21,24]), the behavior of the target during the decision steps (static in [8,16,21,22,24,25] and dynamic in [11–13]), and the number of steps in the planning horizon (one in [8,16,21], two in [11] and *N* in [12,13,22,24,25]). An additional difference, which is especially relevant to this work, is the type of detection sensorial model used to test the performance of these approaches. The observation models under test belong to the following groups: binary 0/1 detection probability outside/inside the observed cell in [12,13,17] and outside/inside the visible area in [25], stepped detection probability with zero/constant probability outside/inside the observed cell in [11], and different continuous differentiable distance-based detection probability functions that approximate the behavior of radar detection functions in [8,21,22,24]. Therefore, all these approaches are tested with streamlined observation models that do not present the richness of behaviors that appear in many sensors and that usually facilitate the search form the optimization algorithm point of view.

This work tackles the MTS problem following the last option, implementing the multi-agent expected time of detection as the utility function and CEO as the optimization algorithm. Therefore, it combines one of the two utility functions presented in [13] for the multi-agent MTS and the optimization method used in [12] for the single-agent one. We only test ET in the controller because the work in [13] showed that the results with ET are usually no worst than the results with the discounted probability of detection. Besides, we prefer CEO to the BOA used in [13] due to the significant smaller computational time of CEO, although the results in [13] suggest that BOA usually improves ET further than a technique whose results can be extrapolated to CEO. In fact, [13] shows that the ET obtained with a BOA where the action sequence probability *q^l^* is estimated with a Bayesian Network (BN [45]) with dependencies among the variables within the action sequence are better than those obtained with a BOA using a BN without dependencies, whose *q^l^* is estimated almost as the *q^l^* in CEO.

Finally, it is important to highlight that one of the most relevant differences between this work and the others is the analysis of the performance of the system with the complex detection sensor models presented in the following section. In this respect, this paper extends the study of the validity of the elements (utility function and optimization method) of the systems presented in [12,13], tested in those works only with the ideal binary 0/1 probability sensor which only and always detects the target when the agent and target coincide (*i.e.*, $P(z_{i}^{k}=D|\tau^{k},s_{i}^{k})$ equals 1 when $\tau^{k}=s_{i}^{k}$ and 0 otherwise), to other types of sensors.

## 4. Experimental Setups

This section presents the mathematical formulation of the three MTS tasks that are used in the paper to test the performance of the ET-CEO-based autonomous and intelligent system described in Section 2.2. Each tasks constitutes an experimental setup, and always differ from the rest in its observation probability model and sometimes in the dimensionality of the search/action space. The observation models under test are associated to a radar working in a space free of obstacles, an ultrasonic scanner working in a structured environment, and a camera and vision identification system working in an orientation-dependent search space. The search space in the first two tasks is a discretized (*x*, *y*) bidimensional grid, while in the third is a discretized (*x*, *y*, *θ*) tridimensional grid. The action space in the first and third task are the eight cardinal directions (high level actions obtained by the controller layer and used by the agents low level embedded guidance and control system to drive the agents towards the next cell/sensing position), while in the second it is restricted by the map of the environment to the valid directions in each cell. These choices let us test the performance of the approach under different circumstances. The first sensor is modeled either with a binary 0/constant detection probability outside/inside a visible area or with a realistic continuous differentiable distance-based detection probability to analyze how the ET-CEO approach works with the types of models used by the closely related solutions [8,11,21,22,24]. The second sensor is modeled with a complex function that only presents a smooth decreasing behavior over the valid range of the sensor which is not occluded by the static objects of the environment map. Therefore, the second experimental setup permits us to verity if the ET-CEO approach is able to deal with a non-differentiable sensor that presents a different behavior over different cells of the search space and with a different action space over each cell. Finally, the third sensor is modeled with a multiform function dependent on the distance and relative orientation between the agent and target, which presents a smooth decreasing behavior on the distance and a non-differentiable combed form within the camera field of view on the relative orientation. Hence, the third experimental setup permits us to validate the performance of the ET-CEO approach in an orientation dependent space with a really complex sensor.

In the following, we formalize in detail the different experimental setups and the probability observation models used within them.

### 4.1. Searching a Life-Raft in the Sea Using a Radar

In the first experimental setup, the agents are a group of Unmanned Air Vehicles (UAVs) and the target is a life-raft that has to be find in the sea [21]. Each UAV is equipped with (1) a Global Positioning System (GPS) receiver that provides accurate information about the UAV location and (2) a downward looking primary radar used to detect the signal returned by a radar reflector mounted on the life-raft. In order to model this MTS task, we assume that:

- Ω (the space where the target is contained) is a two dimensional grid whose cells represent adjacent square regions of the sea surface, *τ_k_* (the target state) is the cell where the life-raft is located, Δ (the action space) consists on the eight high level actions associated to the cardinal directions (North, North East, East, South East, South, South West, West, North West; *i.e.*, N,NE,E,SE,S,SW,W,NW) that make the UAVs flight at a fixed height *h* from the central location of one cell to the central location of one of its adjacent cells, $s_{i}^{k}$ (each agent state) is defined by the UAV ( $x_{i}^{k}$, $y_{i}^{k}$, *h*)-location, and *ϵ* (the environment state) is associated to the radar parameters and the wind and sea currents;
- The initial belief *b*(*τ*^0^), which represents the probability of finding the life-raft in each cell of the grid used to discretize the sea searching region, is built with information of a ship-wreck. *P*(*τ^k^*|*τ^k^*^+1^, *ϵ*), which constitutes the life-raft transition/displacement model, is built accordingly to the wind and sea currents in the searching region. The scenarios under test in Section 5.1 are defined by different tuples of both probability functions and initial UAVs locations (*i.e.*,{*b*(*τ*^0^),*P*(*τ^k^*|*τ^k^*^+1^, *ϵ*), $s_{1:M}^{0}$ });
- We model the radar detection likelihood, $P(z_{i}^{k}=D|\tau^{k},s_{i}^{k},\epsilon )$, using one of the two following probability functions:
  – The first model, presented in Equation (7), shows a complex non-linear dependency of the detection likelihood with the Signal to Noise Ratio (*S_NR_*) and the Threshold to Noise Ratio (*T_NR_*). The selected expression is derived in [46] taking into account different properties of the target and noise signals in the radar. It is valid for reflectors with Swerling 3 Radar Cross Section (RCS) [47,48], because the radar reflector mounted on the life-raft behaves as a primary scatter with several smaller ones. According to Equations (8) and (9), the *S_NR_* is inversely proportional to the distance $d_{\tau ,i}^{k}$ between the target ( $x_{\tau }^{k}$, $y_{\tau }^{k}$, 0) and UAV ( $x_{i}^{k}$, $y_{i}^{k}$, *h*) location, and *T_NR_* can be calculated given the desired probability of false alarm of the radar *P_fa_*. Finally, the parameter *C_ϵ_* in Equation (8) englobes multiple properties of the radar (signal and noise power, directivity gains, wavelength, *etc*.) and can be obtained from Equation (7) using the bisection numerical method [49] and fixing the detection likelihood $P(z_{i}^{k}=D|\tau^{k},s_{i}^{k},\epsilon )$ for a given distance $d_{\tau ,i}^{k}$ and the probability of false alarm *P_fa_*.
    (7) $$P(z_{i}^{k}=D|\tau^{k},s_{i}^{k},\epsilon )=\left(1+\frac{2\cdot S_{NR}\cdot T_{NR}}{{\left(2+S_{NR}\right)}^{2}}\right)e^{-2\cdot T_{NR}/(2+S_{NR})}$$
    (8) $$\text{with}\;S_{NR}=\frac{C_{\epsilon }}{{\left(d_{\tau ,i}^{k}\right)}^{4}}$$
    (9) $$T_{NR}=-\log(P_{fa})$$
    (10) $$d_{\tau ,i}^{k}=\sqrt{{\left(h\right)}^{2}+{\left(x_{i}^{k}-x_{\tau }^{k}\right)}^{2}+{\left(y_{i}^{k}-y_{\tau }^{k}\right)}^{2}}$$
  – The second model, presented in Equation (11), shows an ideal behavior of a radar that has a high probability *P_d_* of detecting the target up to a fixed distance *δ* between the target and the UAVs, and a null probability after it. The distance $d_{\tau ,i}^{k}$ is also obtained with Equation (10).
    (11) $$P(z_{i}^{k}=D|\tau^{k},s_{i}^{k},\epsilon )=\{\begin{array}{cc} P_{d} & \text{if}\;d_{\tau ,i}^{k}<\delta \\ 0 & \text{otherwise} \end{array}$$

**Figure 2.**
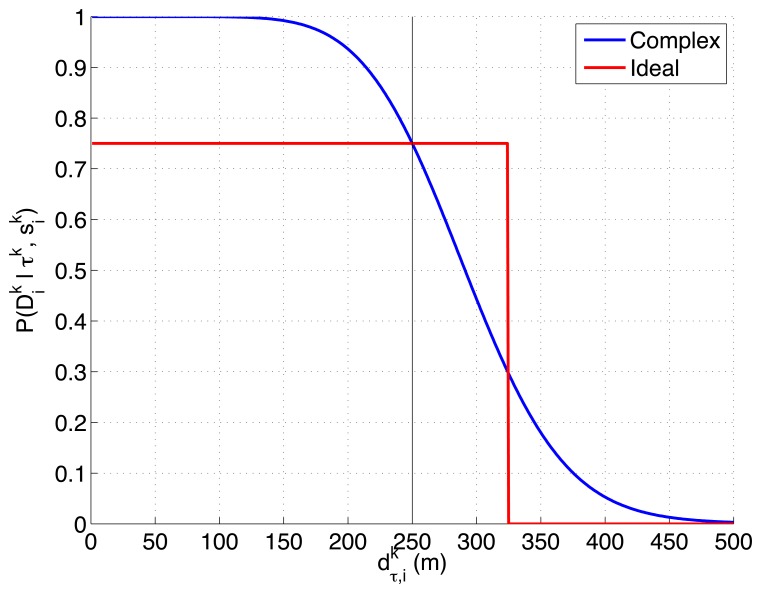
Radar detection probability curves, with the first *complex* model in blue and the second *ideal* model in red, when *P_fa_* = 10^−6^, *C_ϵ_* = 1.126 × 10^11^ (obtained to make $P(z_{i}^{k}=D|\tau^{k},s_{i}^{k},\in )=0.75$ at $d_{\tau ,i}^{k}=h=250\;\text{m}$), *P_d_* = 0.75 and *δ* = 325 m. With UAVs flying at *h* = 250 m, the probability values used in the experiments are placed after the vertical black line.

There are several reasons to test the previous two detection likelihoods, represented in Figure 2, in this experimental setup. On one hand, the first function models the detection performance of a real radar and reflecting element in a free space without occlusions, and its shape (smooth, monotonically decreasing, almost flat initially with a later deep slope) appears in different active sensors (e.g., radars and sonars). On the other hand, the second function is a generalization (consistent on letting the second radar model see multiple consecutive cells and detect the target with a constant probability *P_d_* ≠ 1) of the ideal sensor, with binary 0/1 probability outside/inside the overflown cell, that was used to test our solution to the MTS problem in [12,13]. Therefore, the first choice lets us test the ET-CEO-based autonomous and intelligent system in a realistic common setup, while the second lets us compare the behavior of the first choice against an adapted version of the previously tested ideal sensor model.

Finally, we want to point out that although this experimental setup contains the same elements as the one presented in [21], we model the behavior of the agents, target and radar with different functions. While their radar detection model considers that $P(z_{i}^{k}=D|\tau^{k},s_{i}^{k},\epsilon )\propto \frac{1}{{\left(d_{\tau ,i}^{k}\right)}^{4}}$ (a valid approximation when the radar is working in the deep slope region) our model presents a more realistic behavior in spite of the fact of having fixed its parameters to match the working range of the radar in [21]. Additionally, the system in [21] considers a unique UAV whose controller maximizes the detection probability instead of minimizing the expected time.

### 4.2. Searching a Known Object in a Structured Environment with an Ultrasonic Scanner

In the second experimental setup, the agents are a group of robots and the target is a known object that has to be found inside a building. The robots and the building are equipped with a beaconing system that provides accurate information about the robot locations. Each robot has an ultrasonic scanner used to emit and detect the signal returned by the different objects in the environment. In order to model this MTS task, we assume that:

- Ω is a two dimensional grid that discretizes, using square cells, the building areas under search; τ*_k_* is the location/cell of the known object within the grid, Δ contains again the eight actions associated to the cardinal directions (N,NE,E,SE,S,SW,W,NW) that make the robot move from the central location of one cell to the central location of its adjacent cells; $s_{i}^{k}$ is the robot ( $x_{i}^{k}$, $y_{i}^{k}$)-location; and the environment variables *ϵ* = {*S_ϵ_*,*map_ϵ_*} represent the set of sonar parameters (*S_ϵ_* = {*A_ϵ_*, *B_ϵ_*}, with *A_ϵ_* = {*C_ϵ_*, *P_fa_*} and *B_ϵ_* = {*δ_min_*, *δ_max_*,*β*}) and the map (*map_ϵ_*) of the building, which shows which cells of the grid are occupied by static objects.
- The initial belief *b*(*τ*^0^) represents the probability of finding the known object in each cell of the grid and is built with a priori information of the usual object locations; and *P*(*τ^k^*|*τ^k^*^+1^, *map_ϵ_*) is the object displacement model built considering the usual movements of the object and the physical restrictions imposed by the static objects of the map.
- The ultrasonic scanner detection likelihood $P(z_{i}^{k}=D|\tau^{k},s_{i}^{k},\epsilon )$ is defined with Equation (12). It combines the effects of the three behaviors modeled with the probability functions *P_f_*_1_(·), *P_f_*_2_(·) and *P_f_*_3_(·). The first, *P_f_*_1_(·), corresponds to the detection likelihood model of an active sensor in free space without occlusions. Indeed, we use the expression of an active radar when the reflecting element has a Swerling 1 RCS [46–48] to model the sonar behavior in free space, by setting the values of its parameters to make it work in the sonar detection range, because it is simpler that Equation (7) and matches better the behavior of a generic object with multiple scattering surfaces. The second, *P_f_*_2_(·), models the occluding behavior of the static elements *obj_l_* in the map, which eliminate any probability of seeing the object when any of them is placed in the Line Of Sight (LOS) between the target *τ^k^* and the robot $s_{i}^{k}$. Finally, *P_f_*_3_(·) is used to model the working range of the sonar [*δ_min_*, *δ_max_*] and the existence of other unexpected objects. Objects placed closer than *δ_min_* are not detected because that locations are associated with the period of time after the emission of the signal where the sensor is off to avoid detecting the emitted signal directly. Objects placed at bigger distances that *δ_max_* are ignored to reduce the effects produced by the existence of multi-path reflections. The existence of unexpected objects is modeled, inspired by the sensor error model presented in [50], as a decreasing linear function of the distance under the assumption that unexpected objects can appear suddenly in any position of the space with the same probability. Therefore as the distance of the object under search grows, there is a smaller linear probability of detecting it because the number of unexpected objects in between increases. In other words, the decreasing parameter *β* is associated with the probability of the appearance of unexpected objects.
  (12) $$P(z_{i}^{k}=D|\tau^{k},s_{i}^{k},\epsilon )=P_{f1}(\tau^{k},s_{i}^{k},A_{\epsilon })\cdot P_{f2}(\tau^{k},s_{i}^{k},{\text{map}}_{\epsilon })\cdot P_{f3}(\tau^{k},s_{i}^{k},B_{\epsilon })$$
  with
  (13) $$P_{f1}(\tau^{k},s_{i}^{k},A_{\epsilon })=e^{-T_{NR}/\left(S_{NR}+1\right)}$$
  (14) $$P_{f2}(\tau^{k},s_{i}^{k},{\text{map}}_{\epsilon })=\{\begin{array}{ll} 0 & \text{if}\exists obj_{l}\;\in \;map_{ɛ}\;intersect(LOS(\tau^{k},s_{i}^{k}),obj_{l})=true\\ 1 & \text{otherwise} \end{array}$$
  (15) $$P_{f3}(\tau^{k},s_{i}^{k},B_{\epsilon })=\{\begin{array}{ll} 1-\beta \cdot d_{\tau ,i}^{k} & \text{if}\;\delta_{min}<d_{\tau ,i}^{k}<\delta_{max}\\ 0 & \text{otherwise} \end{array}$$
  (16) $$S_{NR}=\frac{C_{\epsilon }}{{\left(d_{\tau ,i}^{k}\right)}^{4}}$$
  (17) $$T_{NR}=-\log(p_{fa})$$
  (18) $$d_{\tau ,i}^{k}=\sqrt{{\left(x_{i}^{k}-x_{\tau }^{k}\right)}^{2}+{\left(y_{i}^{k}-y_{\tau }^{k}\right)}^{2}}$$

**Figure 3.**
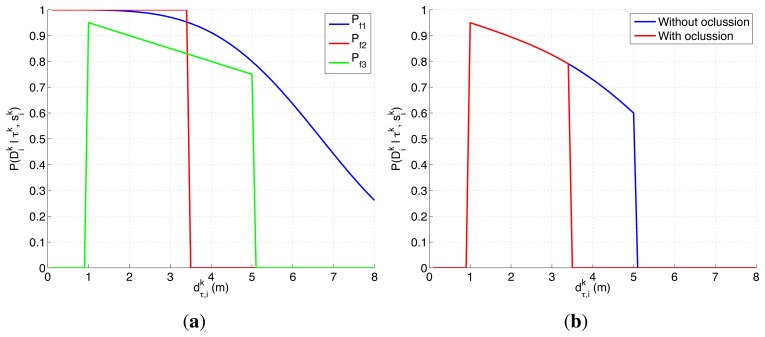
Ultrasonic scanner detection probability curves when *P_fa_* = 10^−6^,*C_ϵ_* = 3.81 × 10^4^ (obtained to make *P_f_*_1_(·) = 0.8 at $d_{\tau ,i}^{k}=5\;\text{m}$), there is an occluding object at 3.5 m of the agent, *δ_min_* = 1 m, *δ_max_* = 5 m, and *β* = 0.05. While Figure 3a shows the composing curves, Figure 3b shows the detection probability with and without the occluding object in the LOS. (**a**) Composing probability functions; (**b**) Ultrasonic detection likelihood.

There are several reasons that make the experimental setup of this section harder than the presented in the previous one. On one hand, the ultrasonic scanner likelihood, represented in Figure 3, is a complex model that presents a smooth decreasing behavior over the valid range of the sensor when the structure of the environment permits it and a different behavior, originated by the occlusive objects of the map, over the different locations of the space Ω. On the other hand, not only the objects of the map affect the target belief (through the dependency of the the target transition and sensor models on the map), but they also restrict the possible agents movements to cells without known objects. Therefore, for this experimental setup we need to incorporate some of the usual ways to handle constraints in optimization problems, such as including the constraints in the utility function [51–53], penalizing the objective function value of the unfeasible solutions [54] or using searching strategies capable of only generating valid solutions [55]. In this case, we use the two last approaches, implemented as an:

(1) Adapted Sampling (AS) step of CEO that only generates valid solutions. This sampling step, instead of sampling the values of each action variable $u_{i}^{k+j}$ independently from each column in the action probability table *q^l^*, it samples the values in a sequence with increasing time step *k* + *j*, from the probability obtained after momentarily canceling, in the column related to the corresponding variable, the values of the rows of those actions that will make the robot move from its current position to an occupied one. That is, while sampling the new possible solutions, we only generate those actions that are allowed in each time step given the previous time step and the initial location, using the probability action sequence *q^l^* sequentially modified accordingly to the map restrictions.
(2) Additive term in the ET evaluation that penalizes those solutions that overpass a cell occupied by a known object (*i.e.*, calculating the new ET as ET_Algorithm 2_+N*PT_#_( $s_{1:M}^{k+1:k+N}$)). We implement/test the following Penalization Terms PT_#_(·): the number of occupied/non valid cells of the UAVs trajectories (PT_1_), the number of cells to reach the last step after the first non valid cell (PT_2_), and the number of non-valid cells plus non-valid continuous cells (PT_3_). The penalization is multiplied by N before adding it to the ET obtained by Algorithm 2 to ensure that any solution which does not overpass a forbidden cell (including the solution whose ET_Algorithm 2_ = *N* because it does not collect any probability of locating the target) is better than any solution that overpass a non valid cell.

As a whole, this experimental setup lets us test the ET-CEO-based autonomous and intelligent system with a complex sensor in a constrained structured environment.

### 4.3. Searching a Flat Object in an Orientation-Dependent Scenario with a Camera

In the third experimental setup, the agents, looking for a flat object in a space without obstacles, are equipped with a positioning systems that provides accurate information about each agent location and orientation, and with a camera and vision-based processing system that is able to identify the target. We have decided to exclude the presence of occluding objects in this experimental setup to show how other new elements of the sensor model affect the searching capabilities of the MTS system under test. In order to model this MTS task, we make the following assumptions:

- Ω is a three dimensional grid that discretizes, using cubic cells, the space of possible (*x*, *y*, *θ*)-positions of the target; *τ_k_* is the (*x*, *y*, *θ*) location/cell of the known object within the grid, A contains again the eight actions associated to the cardinal directions (N,NE,E,SE,S,SW,W,NW) that make the agent move from the central location of one cell to the central location of its adjacent cells with the orientation defined by the cardinal action; $s_{i}^{k}$ is the agent ( $x_{i}^{k}$, $y_{i}^{k}$, $\theta_{i}^{k}$)-position; and the environment variables *ϵ* = {*A_ϵ_*, *θ_ϵ_*, *λ_ϵ_*} are related with the area of the object under search, the camera field of view, and the effectiveness of the vision based identification algorithm. Finally, we want to highlight that the orientation *θ* of the flat object is defined by the orientation of the vector normal to its surface.
- The initial belief *b*(*τ*^0^) represents the probability of finding the known object in each cell of the three dimensional grid and is built with a priori information of the usual object locations and orientations. *P*(*τ^k^*|*τ^k^*^+1^) is the object displacement model built considering the expected object displacements in the three dimensions of the search space.
- The detection likelihood $P(z_{i}^{k}=D|\tau^{k},s_{i}^{k},\epsilon )$ of the camera and vision identification system is defined with Equation (19). It combines the effects of the behaviors modeled with the probability functions *P_f_*_4_(·) and *P_f_*_5_(·). The first function, *P_f_*_4_(·), models the variability of the detection probability of the camera and vision identification system with respect the distance and the relative orientation that exist between the object and the agent. This model is obtained under several assumptions. The first hypothesis is the dependency of the detection probability of the vision system on the effective surface *S* of the object over the camera image. This dependency, collected in Equation (20), makes the detection probability of the vision system grow with the increment of the size of the object in the captured image. The second premise, stated in Equation (22), is the variation of the size of the object of the image (1) with the inverse of the square of the distance of the object ( $\propto A_{\in }/{\left(d_{\tau ,i}^{k}\right)}^{2}$) and (2) with the absolute value of the cosine of relative orientation of the normal vector to the surface of the object with respect the agent ( $\left|\cos(\theta_{\tau }^{k}-\theta_{i}^{k})\right|$), under the assumption that the object is, ideally, flat and equally identifiable for both sides. This makes a flat object parallel to the camera (that has a normal vector perpendicular to it) invisible, inducing an abrupt behavior (that let us test the performance of the autonomous and intelligent system under extreme conditions) in the probability detection model at certain orientation. The second function, *P_f_*_5_(·), models the visibility restrictions and the camera field of view *θ_ϵ_* imposed by the camera field of view, which eliminate any probability of seeing an object placed outside the region (*RegionInFOV*) defined by the agent location and orientation ( $x_{i}^{k}$, $y_{i}^{k}$, $\theta_{i}^{k}$). If required, the inclusion of additional visual constraints (such as the valid distance ranges associate to the camera depth of field or to the occluding objects of a static map) or the behavior of other types of visible-orientable-dependent objects can be formulated within Equation (21) and Equation (22).
  (19) $$P(z_{i}^{k}=D|\tau^{k},s_{i}^{k},\epsilon )=P_{f4}(\tau^{k},s_{i}^{k},A_{\epsilon },\lambda_{\epsilon })\cdot P_{f5}(\tau^{k},s_{i}^{k},\theta_{\epsilon })$$
  with
  (20) $$P_{f4}(\tau^{k},s_{i}^{k},A_{\epsilon },\lambda_{\epsilon })=(1-e^{-\lambda_{\epsilon }S})$$
  (21) $$P_{f5}(\tau^{k},s_{i}^{k},\theta_{\epsilon })=\{\begin{array}{ll} 1 & \text{if}\;(x_{\tau }^{k},y_{\tau }^{k})\in \;\text{RegionInFOV}(x_{i}^{k},y_{i}^{k},\theta_{i}^{k},\theta_{\epsilon })\\ 0 & \text{otherwise} \end{array}$$
  (22) $$S=\frac{A_{\epsilon }}{{\left(d_{\tau ,i}^{k}\right)}^{2}}\left|cos(\theta_{\tau }^{k}-\theta_{i}^{k})\right|$$
  (23) $$d_{\tau ,i}^{k}=\sqrt{{\left(x_{i}^{k}-x_{\tau }^{k}\right)}^{2}+{\left(y_{i}^{k}-y_{\tau }^{k}\right)}^{2}}$$

**Figure 4.**
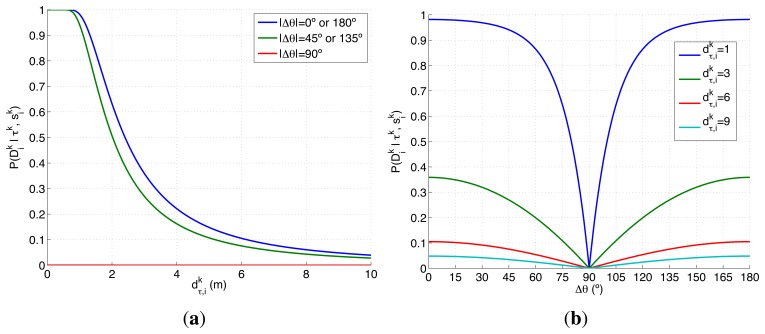
Detection probability curves of the camera and vision identification system when *A_ϵ_* = 1, *λ_ϵ_* = 4 and the target is placed within the camera field of view *θ_ϵ_* = 60. (**a**) Dependency on $d_{\tau ,i}^{k}$. It shows the detection probability curves of different relative angles $\Delta \theta =\theta_{\tau }^{k}-\theta_{i}^{k}$; (**b**) Dependency on $\Delta \theta =\theta_{\tau }^{k}-\theta_{i}^{k}$. It shows the detection probability curves of different distances $d_{\tau ,i}^{k}$.

There are several reasons that make the experimental setup of this section harder than the presented in the previous ones. On one hand, the search space Ω has a third dimension, the orientation, that is not considered in the previous experimental setups. This has two important consequences. First, the new dimension has to be considered in the initialization of the belief space and in the definition of the target transition function. Second, it increases the cost of the evaluation of the RBF prediction step and of the ET function. On the other hand, the detection likelihood of the camera and vision identification system, represented in Figure 4, is a complex two dimensional model that presents, within the camera field of view, a smooth decreasing shape for the distance and a damped combed behavior for the relative orientation of the target with respect to the agent. Additionally, this sensor is not omnidirectional like the ones selected in the previous experimental setups. Therefore, this experimental setup lets us test the ET-CEO-based MTS autonomous and intelligent system in a space with higher dimensionality with a especially complex sensor.

Finally, it is worth noting that when the initial probability target in each possible ( $x_{\tau }^{0}$, $y_{\tau }^{0}$) location and the target transition probability are not dependent on the orientation, the problem has an unnecessary additional dimension. To reduce it, we can use a detection likelihood which is not dependent on the orientation. The likelihood model of the reduced dimensional system can be obtained, applying the marginalization and Bayes rule, as $P(z_{i}^{k}=D|\tau^{k},s_{i}^{k},\epsilon )=\int_{\theta_{\tau }^{k}}P(\tau^{k})P_{f4}(\tau^{k},s_{i}^{k},A_{\in },\alpha_{\in })\cdot P_{f5}(\tau^{k},s_{i}^{k},\theta_{\in })d\theta_{\tau }^{k}$.

## 5. Results and Discussion

In this section we show and discuss the results obtained by the ET-CEO autonomous and intelligent system presented in Section 2 under different scenarios of the experimental setups presented in Section 4. Each experimental setup has the fixed target state space Ω, action space Δ, observation probability model $P(z_{i}^{k}|\tau^{k},s_{i}^{k},\epsilon )$ and deterministic agent behavior described in Sections 4.1, 4.2 or 4.3; while each scenario is deFIned by the initial agents states ( $s_{1:M}^{0}$), the initial target belief *b*(*τ*^0^), the target transition probabilistic model *P*(*τ^k^*|*τ^k^*^+1^, *ϵ*), and, in the second experimental setup, the obstacles map *map_ϵ_*.

We test the complete system in each experiment setup against a static scenario and a dynamic one on 20 independent simulations. The static scenarios (which are special cases of the dynamic ones, where *P*(*τ^k^*|*τ^k^*^−1^) equals 1 when *τ^k^* =*τ^k^*^−1^ and 0 otherwise) are provided to visualize more easily (isolatedly from any transition model) the effects of the sensor models in the MTS system. The dynamic scenarios are constructed to be able to analyze the performance of the MTS system in generic dynamic cases. All of them have been carefully selected to show the influence of the different sensor models under different circumstances.

In order to characterize statistically the results of the 20 simulations run over each experimental setup and scenario, we fix the values of the measurements $z_{i}^{k}$ obtained by the agents during the whole simulation to its no-detection value. The purpose of this choice is to isolate the variability of the results of the simulations caused by the ET-CEO controller (associated to the non-deterministic/heuristic nature of the CEO) from the variability (eliminated by making $z_{i}^{k}=\bar{D}$) originated by the measurements $z_{i}^{k}$ obtained during each simulation (due to the direct relation that exists between the results of the data fusion layer and the actual measurements, and the interactions that appear between the results of the data fusion and controller layers). Therefore, the results of this article, independent on the values of the measurements that should be obtained in each simulation, are the corresponding to the worst possible case that occurs while the sensors do not detect the target during the whole planning process.

Other common characteristics of all the simulations are the horizon length *N* = 10 and the following CEO parameters: percentil of best solution *ϰ* = 0.01, smoothing factor *α* = 0.6, and maximum number of iterations equal to 20. Additionally, in all the simulations we fix the number of samples accordingly to the number of agents *M,* number of steps in the controller horizon *N* and number of actions |Δ| using the following expression: *E* = 10 · *M* · *N* · |Δ|.

**Figure 5.**
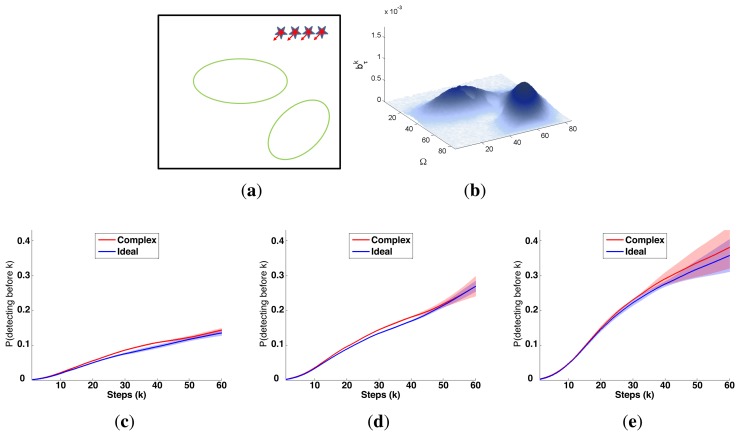
Properties and Information Gain (IG) curves of the static scenario of the radar experimental setup, (**a**) Schema; (**b**) Initial belief *b*(*τ*^0^); (**c**) IG, *M* = 1; (**d**) IG, *M* = 2; (**e**) IG, *M* = 4.

**Figure 6.**
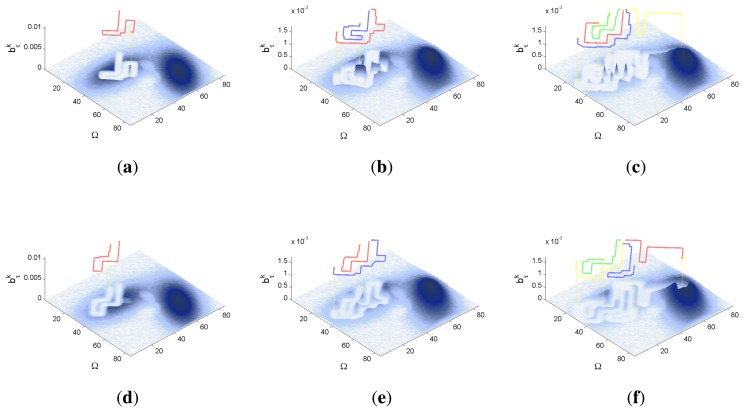
Simulations in the static radar scenario. (**a**) Ideal, *M* = 1; (**b**) Ideal, *M* = 2; (**c**) Ideal, *M* = 4; (**d**) Complex, *M* = 1; (**e**) Complex, *M* = 2; (**f**) Complex, *M* = 4.

**Figure 7.**
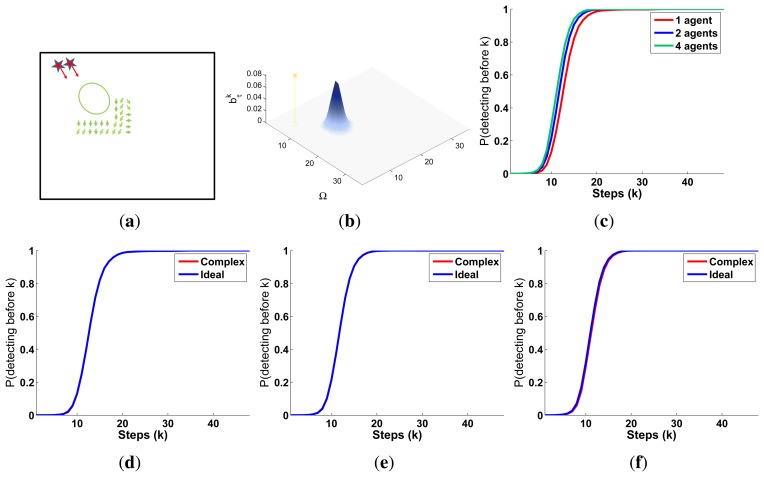
Properties and IG curves of the dynamic scenario of the radar experimental setup. (**a**) Schema; (**b**) Initial belief *b*(*τ*^0^); (**c**) IG, Complex; (**d**) IG, *M* = 1; (**e**) IG, *M* = 2; (**f**) IG, *M* = 4.

**Figure 8.**
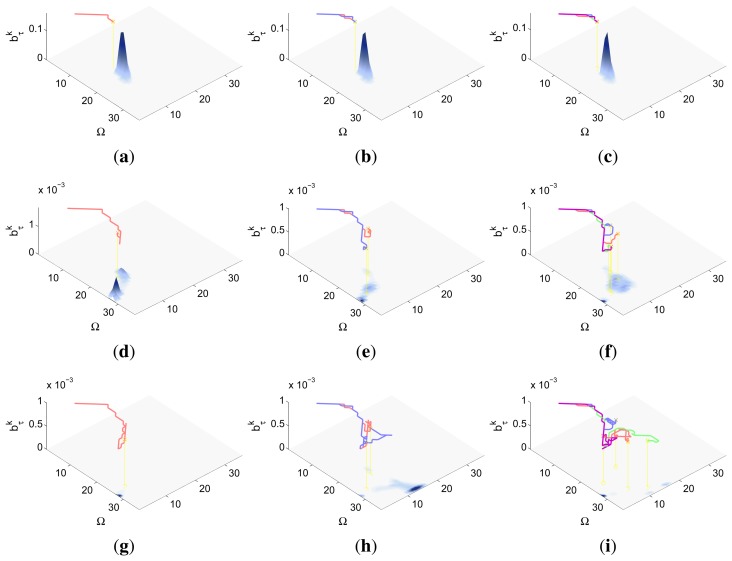
Simulations with the ideal sensor in the dynamic radar scenario.(**a**) *k* = 10, *M* = 1; (**b**) *k* = 10, *M* = 2; (**c**) *k* = 10, *M* = 4; (**d**) *k* = 25, *M* = 1; (**e**) *k* = *25, M* = 2; (**f**) *k* = *25, M* = 4; (**g**) *k* = *50, M* = 1; (**h**) *k* = 50, *M* = 2; (**i**) *k* = 50, *M* = 4.

**Figure 9.**
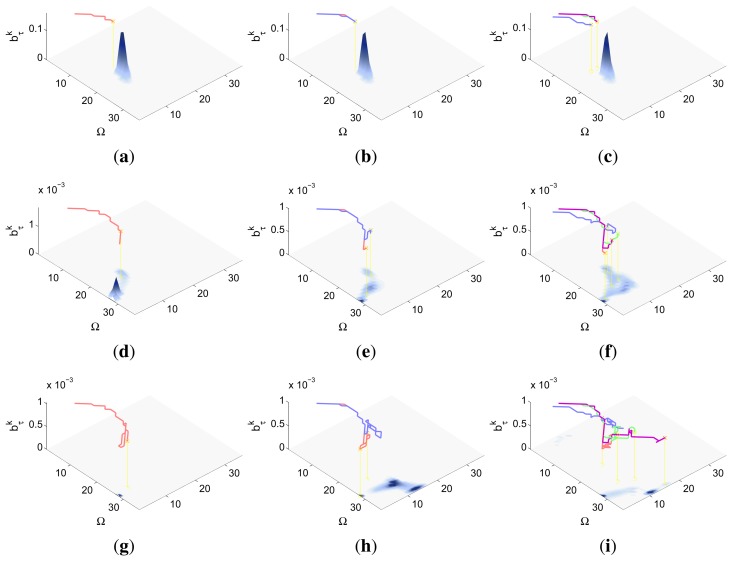
Simulations with the complex sensor in the dynamic radar scenario.(**a**) *k* = 10, *M* = 1; (**b**) *k* = 10, *M* = 2; (**c**) *k* = 10, *M* = 4; (**d**) *k* = 25, *M* = 1;(**e**) *k* = 25, *M* = 2; (**f**) *k* = 25, *M* = 4; (**g**) *k* = 50, *M* = 1; (**h**) *k* = 50, *M* = 2; (**i**) *k* = 50, *M* = 4.

**Figure 10.**
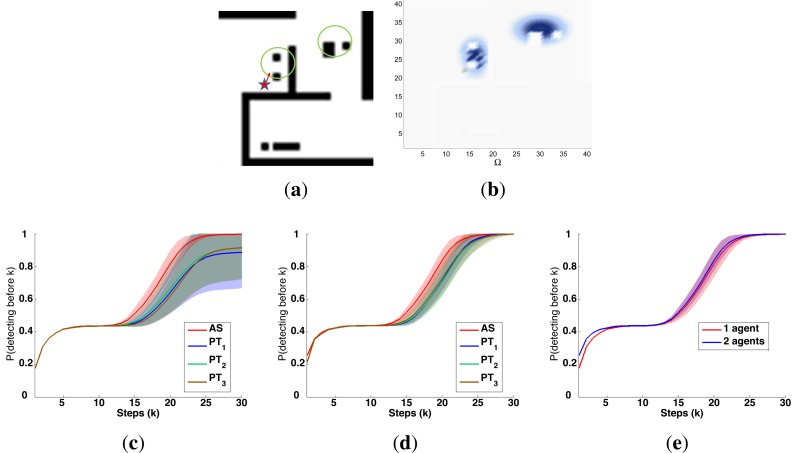
Properties and IG curves of the static ultrasonic scanner experimental setup. (**a**) Schema; (**b**) Initial belief *b*(*τ*^0^); (**c**) IG, *M* = 1; (**d**) IG, *M* = 2; (**e**) IG, AS.

**Figure 11.**
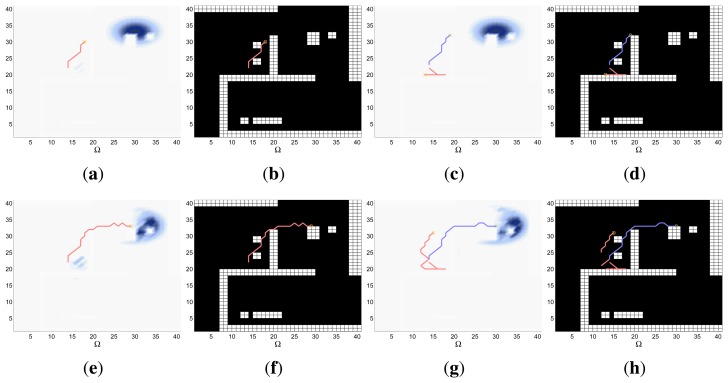
Simulations in the static ultrasonic scanner scenario. (**a**) *k* = 10, *M* = 1; (**b**) *k* = 10, *M* = 1; (**c**) *k* = 10, *M* = 2; (**d**) *k* = 10, *M* = 2; (**e**) *k* = 20, *M* = 1; (**f**) *k* = 20, *M* = 1; (**g**) *k* = 20, *M* = 2; (**h**) *k* = 20, *M* = 2.

**Figure 12.**
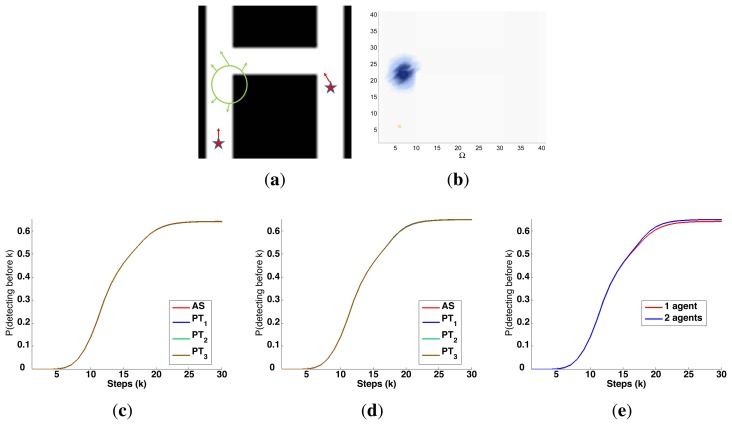
Properties and IG curves of the dynamic ultrasonic scanner experimental setup. (**a**) Schema; (**b**) Initial *b*(*τ*^0^); (**c**) IG, *M* = 1; (**d**) IG, *M* = 2; (**e**) IG, Adapted Sampling (AS).

**Figure 13.**
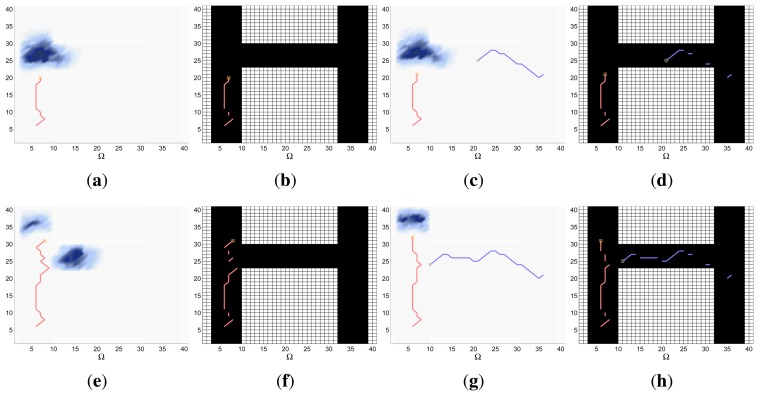
Simulations in the dynamic ultrasonic scanner scenario. (**a**) *k* = 15, *M* = 1; (**b**) *k* = 15, *M* = 1; (**c**) *k* = 15, *M* = 2; (**d**) *k* = 15, *M* = 2; (**e**) *k* = 25, *M* = 1; (**f**) *k* = 25, *M* = 1; (**g**) *k* = 25, *M* = 2; (**h**) *k* = 25, *M* = 2.

**Figure 14.**
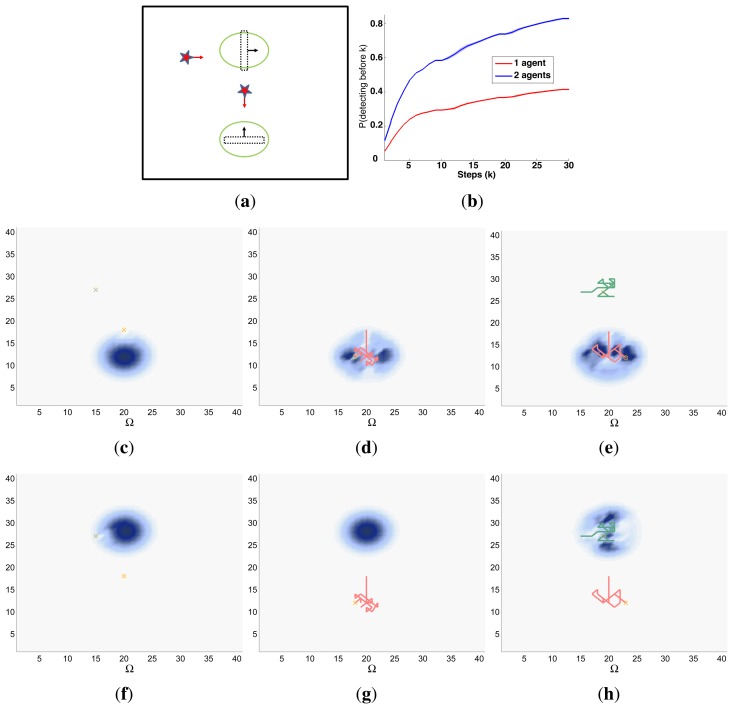
Simulated results in the static camera scenario. (**a**) Scenario; (**b**) IG; (**c**) North, Initial belief *b*(*τ*^0^); (**d**) North, *M* = 1; (**e**) North, *M* = 2; (**f**) East, Initial belief *b*(*τ*^0^); (**g**) East, *M* = 1; (**h**) East, *M* = 2.

**Figure 15.**
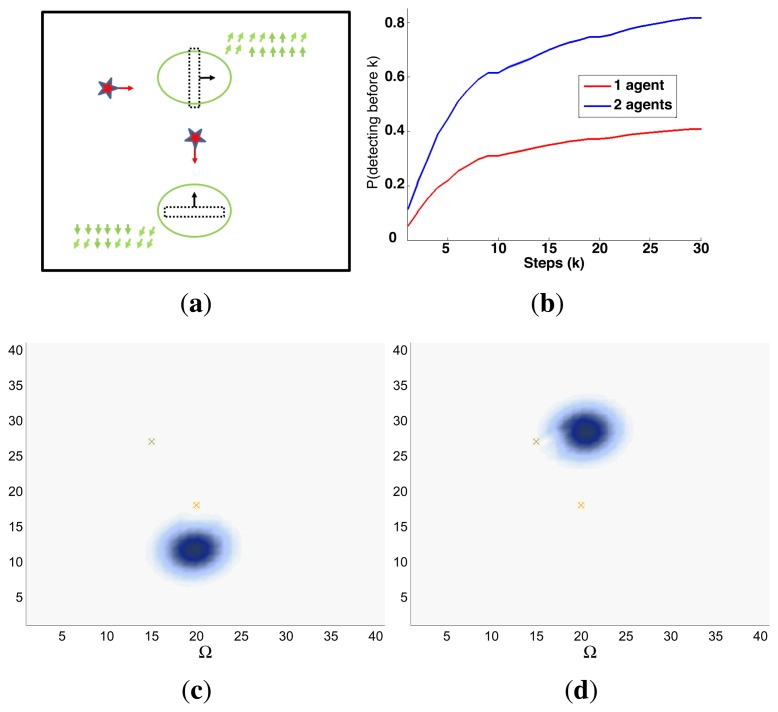
Properties and IG curves of the dynamic camera scenario. (**a**) Scenario; (**b**) IG; (**c**) North, Initial belief *b*(*τ*^0^); (**d**) East, Initial belief *b*(*τ*^0^).

**Figure 16.**
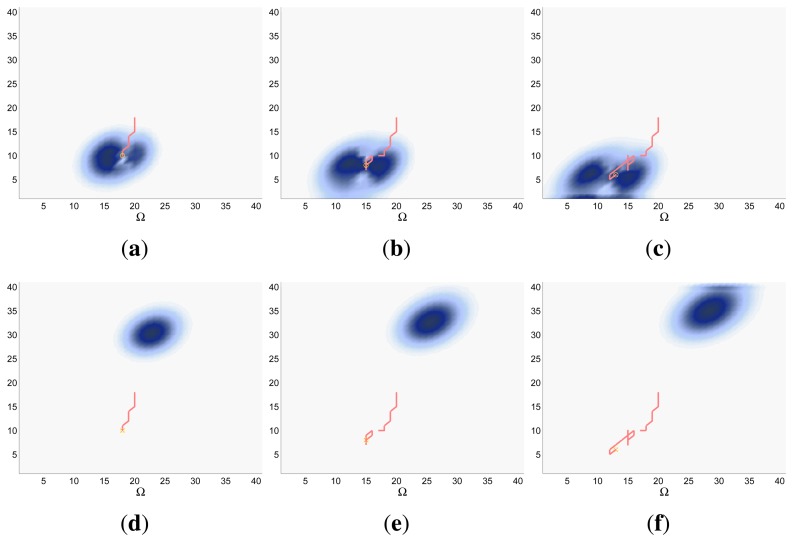
Simulated results in the dynamic camera scenario with *M* = 1 agent. (**a**) North, *k* = 10; (**b**) North, *k* = 20; (**c**) North, *M* = 1, *k* = 30; (**d**) East, *k* = 10; (**e**) East, *k* = 20; (**f**) East, *k* = 30.

**Figure 17.**
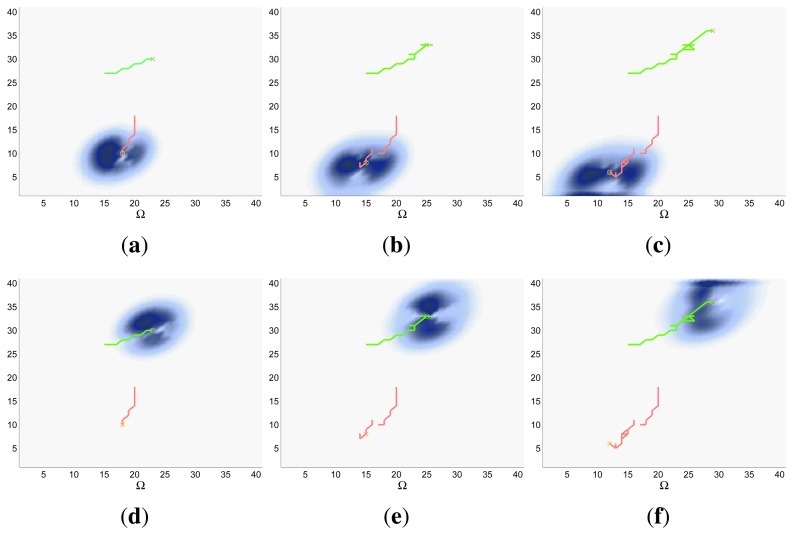
Simulated results in the dynamic camera scenario with *M* = 2 agents. (**a**) North, *k* = 10; (**b**) North, *k* = 20; (**c**) North, *k* = 30; (**d**) East, *k* = 10; (**e**) East, *k* = 20; (**f**) East, *k* = 30.

Finally, the properties of the scenarios of each experimental setup and the results of their associated simulations are analyzed in the three following sections (Sections 5.1–5.3), while the computacional cost of the ET-CEO controller is characterized in Section 5.4 and an overall discussion is presented in Section 5.5. Moreover, the results within Sections 5.1–5.3 are represented in Figures 5, 6, 7, 8, 9, 10, 11, 12, 13, 14, 15, 16 and 17 with the following types of graphics:

- A symbolic representation of the scenario (which appears in the graphics labeled as *Schema* in Figures 5, 7, 10, 12, 14 and 15) where the initial location of the agents is represented with a red star, the probability regions with significative values in the initial belief are framed within green ellipses, and the main components of the target dynamics are represented with green arrows. Besides, in the second setup we also show the map of the environment highlighting in white the cells of the grid that are occupied by static known objects, and in the third setup we include some information related with the target believed orientation.
- The initial target belief (which appears in the graphics labeled as *Initial belief b*(*τ*^0^) in Figures 5, 7, 10, 12, 14 and 15) represented in the first experimental setup as a colored height map, and in the second and third experimental setup as a colored image. In both cases, darker shades represent higher beliefs and lighter ones smaller.
- The Information Gain (IG) or probability of detecting the target before each time step *k*, which is formally defined as $P({\text{U}}_{i=1:M,l=1:k}D_{i}^{l}|s_{1:M}^{1:k},z_{1:M}^{1:k},\epsilon ))$ and can be calculated (see Appendix) as $\left[1-\sum_{\tau^{k}\in \Omega }\prod_{i=1}^{M}P({\bar{D}}_{i}^{k}|\tau^{k},s_{i}^{k},\epsilon )f(\tau^{k})\right]$, where $f(\tau^{k})=\sum_{\tau^{j-1}\in \Omega }P(\tau^{j}|\tau^{k-1})\prod_{i=1}^{M}P({\bar{D}}_{i}^{k-1}|\tau^{k-1},s_{i}^{k-1})f(\tau^{k-1})$ and *f*(*τ*^1^) = ∑*_τ_*_^0^∈Ω_ *P*(*τ*^1^|*τ*^0^,*ϵ*)*b*(*τ*^0^)). We use it to statistically characterize the results of each scenario, because it is directly related with ET through the expression $ET=\sum_{k=1}^{N}\left(1-IG(k)\right)$, which is a straightforward relationship that other indexes such as the entropy do not exhibit. Therefore, sooner increments of the IG curve are associated, if maintained in later time steps, with smaller ET values. The IG curves, which appear in the graphics labeled as *IG* in Figures 5, 7, 10, 12, 14 and 15, show with a solid colored line the mean of the IG value at each time step *k* over the 20 simulations run for each scenario and experimental setup. The IG standard deviation is also represented by the colored translucent shadow around the IG mean. This shadow is not always symmetric around the mean, as we have limited their values by the maximum and minimum IG possible values (*i.e.*, 1.0 and 0.0).
- A combined representation of the agents trajectories and of the updated belief for a representative simulation of each scenario. The labels of these graphics, which appear in Figures 6, 8, 9, 11, 13, 14, 16 and 17, show different information such as the number of agents *M* and the iteration step *k*. The trajectory of each agent is represented with a continuous color line, and the updated belief is shown using the same representation that appears in the corresponding initial belief graphic. Besides, in the first experimental setup, these types of graphics, shown in Figures 6, 8 and 9, present an updated unnormalized belief, to be able to see in the height map how the agents are consuming the belief during the development of the experiment. These effect is not observed in the same way in the graphics in the second and third experimental setup (see Figures 11, 13, 14, 16 and 17), because the flat color map automatically adjusts the shades of the graphics to the existing beliefs. However, and in spite of the representation, the MTS system works as described in Section 2.2.1.: the belief is always normalized in the update step of the RBF
- Finally, and only in the second experimental setup, a combined representation of the agents trajectories for the selected simulation and of the obstacles environment map. These graphics, that only appear in Figures 11 and 13, are used to be able to check easily if the agents positions are restricted to those cells of the grid that are not occupied by a static known object.

### 5.1. Searching a Life-Raft in the Sea Using a Radar

The first experimental setup is defined by a two dimensional grid, where each cell represents a 80 × 80 m square region of the sea. With this discretization and the properties of the radar probability curves represented in Figure 2, the ideal model, which has a reach of 75 m after the UAVs flight altitude *h* = 250 m, can detect, with a probability of 0.75, a life-raft placed in a 3 cells-diameter circular region around the UAV. The complex model, which has a reach of 250 m at the same UAV altitude, is able to detect, with a probability varying from 0.75 to 0.01, a life-raft placed in a 6 cells-diameter circular region around the UAV. Additionally, the ideal model presents an abrupt behavior while the complex model has a smooth shape. Therefore the scenarios analyzed in this experimental setup, which are presented in the following sections, will let us determine how these different properties affect the ET-CEO MTS system.

#### 5.1.1. Static Scenario

The characteristics and results of the static scenario selected for this experimental setup are shown in Figures 5 and 6. According to the graphics represented in Figure 5a,b, the initial uncertainty about the target location is modeled by a non homogenous belief over a target space Ω of 80 × 80 cells. This belief is distributed over two big regions with smooth and abrupt slopes. Besides, the scenario is defined with different numbers of UAVs *(M* = 1, *M* = 2 and *M* = 4) placed outside the region where there is some initial probability of locating the target, and the simulations consists on 60 time steps, divided in 6 controller optimizations of horizon *N* = 10.

The IG graphics for both sensors, grouped according to the number of UAVs (*M*) in each simulation, are presented in Figure 5c–e. Each of these figures shows that the IG mean curve associated to the complex sensor (red solid line) has, during the majority of the time steps *k*, a higher value than the IG mean curve (blue solid line) associated to the ideal sensor for the same number of UAVs. Additionally, if we analyze the IG mean curves presented in the three figures simultaneously, we can observe that the IG mean values are better in the case with *M* = 4, followed by the cases with *M* = 2 and with *M* = 1. This implies that the ET to detect the target obtained by the autonomous and intelligent system with (1) the complex sensor is in average better (smaller) than the obtained with the ideal one and (2) with many agents is in average better than with only a few. The second behavior is usual in the cases where the probability is extended over different regions and the agents are distributed by the controller over them to be able to see the different regions simultaneously. The first behavior is associated to the smooth decreasing probability curve of the complex sensor, which (1) makes the system accumulate a bigger probability of detecting the target; (2) can improve the distribution of the agents over the searching region; and (3) increments the capability of detecting the target at distant locations. The IG standard deviation, represented by the shadows around the solid lines, grows with the time step (*k*) and with the number of UAVs (*M*). This increasing deviation, not associated to the measurements obtained by the UAVs during the simulations because we fix their values to non-detection, is originated by the heuristic and myopic behavior of the ET-CEO MTS approach, which calculates the ET over a fixed horizon of *N* = 10 time steps, only taking into account the subspace of target states reachable from the current agent locations with *N* actions. Therefore, the complete trajectory originated by our controller is a concatenation of myopic solutions, which can be similarly good according to ET at the initial sections of the trajectory and differ, due to distinct final positions of similar ET-valued sections, in the final steps. Incrementing the number of UAVs makes these variability higher, by allowing more combinations of similarly good trajectories with different final positions. Finally, the IG graphics of this scenario usually show a higher end variability in the experiments with the complex sensor than in the experiments with the ideal one. This behavior is associated to the smoothing of the belief induced by the continuous probability curve of the complex sensor that increments the number of similarly good solutions within a fixed horizon (whose final positions will not be necessarily equally good starting points for the next planning iteration).

The graphics in Figure 6 show the UAVs trajectories and updated belief obtained at the final step (*k* = 60) of a representative simulation, using a different row and a different column for the simulations obtained with each sensor type and number of UAVs (*M*). If we compare the graphics associated to the same number of UAVs (placed in the same column) and different sensors we can observe some variations on the UAV trajectories and final belief, which are the responsible of the differences observed in the IG graphics. The differences in the belief for the simulations with *M* = 2 and *M* = 4 UAV are specially interesting. In the simulations with ideal sensors (see Figure 6b,c) there are some abrupt changes in the belief which are originated by the abrupt change of the ideal sensor model. These abrupt changes do not appear in the corresponding simulations with the complex sensors (see Figure 6e,f), because the smooth decreasing behavior of the complex sensor lets it collect the belief more easily. Finally, the simulation with better results is performed with the complex sensor and *M* = 4 agents (see Figure 6f), where the data fusion system is able to collect a bigger quantity of belief due to a higher distribution of the UAVs over the searching region.

#### 5.1.2. Dynamic Scenario

The characteristics and results of the dynamic scenario selected for the radar experimental setup are shown in Figures 7, 8 and 9. According to the graphics presented in Figure 7a,b, the initial uncertainty about the target location is modeled as a gaussian over a target space Ω of 40 × 40 cells and moved using a transition probability function associated to the existing wind and currents in the selected region of the sea. These probability function makes the mass of the probability, placed initially in the upper left corner of the schema move slowly towards its lower left corner. Again, the scenario is simulated with *M* = 1, *M* = 2 and *M* = 4 UAVs, placed close to the initial region of the gaussian initial belief, during 50 time steps, divided in 5 controller optimizations of horizon *N* = 10.

The IG graphics that compare the results of both sensors for different number of UAVs *M*, represented for this case in Figure 7d–f, are really close: the mean IG values almost overlay and the standard deviation is negligible (smaller than 0.01). The similarity of the IG mean between the sensors occurs because the gaussian used to represent the probability mass of the initial belief is not wide enough to let the ET-CEO MST system benefit from the smooth long tail of the complex sensor. The negligible standard deviation is originated by the narrow shape of the gaussian and by the continuous drift of the belief. Both properties drive the UAVs to similar positions, from the ET point of view, at the end of each section of the trajectory and consequently reduce the IG variability of the simulations. Finally, the detail comparison of the IG curves represented for the same sensor and different number of UAVs in Figure 7c revels that the IG of the simulation with the biggest number of UAVs is slightly better (augments earlier) than the IG of the others. Again, the small difference in the IG values among simulations with the same sensor and different number of UAVs is related to the narrow gaussian of the initial belief, which can be easily collected within only a few sensors.

The graphics in Figure 8 show the UAVs trajectories and updated belief of a representative simulation with the ideal sensor at different time steps (*k* = 10, *k* = 25 and *k* = 50), while the graphics in Figure 9 show their counterparts for the complex sensor. In both figures, we organize the results using a different column for the simulation with a different number of UAVs (*M*) and a different row for each of the selected time steps *k*. The trajectories represented in the curves of both figures and the updated belief value show that the system is able to successfully drive the agents to follow the moving remaining probability mass in these simulations. Additionally, in the ideal sensor case (Figure 8), we can observe at time step *k* = 25 that the remaining of the belief of the simulations with *M* = 4 UAVs is slightly smaller than the remaining belief at the same step of the simulations with *M* = 2 UAVs, and the remaining belief of the simulations with *M* = 2 UAVs is slightly smaller than the remaining belief of the simulations with *M* = 1. The same behavior is observed in the simulation with the complex sensor (Figure 8) at the same time step. Besides, the results of all the simulations at time step *k* = 10 are pretty similar in all the cases, and the results at time step *k* = 50 do not show any fixed pattern. All this happens because, at the initial time steps, the UAVs are too far to collect a significant part of the belief, at time step *k* = 25 the simulations with more UAVs benefit from the fact of using more sensors to gather information about the target location, and at the final time steps the belief mass is eliminated either as a consequence of a sensor measurements or of reaching the grid border. Finally, when we compare the graphics in Figure 8 with their corresponding counterpart (*i.e.,* with the graphic with the same number of *M* and *k*) in Figure 9, we do not observe a dominant (better) behavior in any of the sensors. All this confirms the results observed in the IG curves: the behavior of the system improves with the increment of the number of agents and both types of sensors are overall equally good in this scenario.

### 5.2. Searching a Known Object in a Structured Environment with an Ultrasonic Scanner

The second experimental setup is defined by a two dimensional grid, where each cell represents a 1 × 1 m square region within a building. With this discretization and the properties of the ultrasonic scanner probability curves represented in Figure 3, the sensor, which has a coverage between 1 and 5 m when there are not known objects closer, is able to detect, with a probability varying from 0.95 to 0.6, an object placed in the region defined around the robot between two concentric circular regions with 2 cells and 10 cells of diameter. Additionally, the behavior of this sensor is abruptly modified by the existence of a priori known objects in the map. The existence of these objects also restricts the set of feasible actions in their adjacent cells. The restriction is handled by the CEO algorithm by the methods described in Section 4.2: an Adapted Sampling (AS) step in CEO capable of generating automatically feasible solutions or the inclusion of 3 different Penalization Terms (PT_1_, PT_2_ and PT_3_) in the ET evaluation. Therefore, the scenarios analyzed in this experimental setup will let us determine (1) if the ET-CEO MTS system is able to work with a sensor and action space with a changing behavior dependent on an external map; and (2) if the sampling of feasible solutions works better or worst than the penalization of unfeasible ones.

#### 5.2.1. Static Scenario

The characteristics and results of the static scenario selected for this experimental setup are shown in Figures 10 and 11. According to the graphics represented in Figures 10a,b, the map of the building includes several walls and known obstacles, and the initial object location belief defined over a target space Ω of 40 × 40 cells is concentrated in two different regions placed around the obstacles of the map. These two probability regions have an abrupt slope associated to the places of the map with known obstacles, where there is no probability of locating the object. Besides, the scenario is tested with different numbers of robots (*M* = 1 and *M* = 2) placed close to the left high probability region, and with different versions of ET-CEO, each testing one of the restriction handling techniques (AS, PT_1_, PT_2_ and PT_3_). Finally, the simulations consist on 30 time steps, divided in 3 controller optimizations of horizon *N* = 10.

The curves presented in Figure 10c–e compare the IG values of the simulations with the 4 restriction handling approaches and with *M* = 1 and *M* = 2 agents. Figure 10c,d show that the IG mean value and standard deviation in the simulations with an adapted sampling (AS) step are better (higher mean and narrower standard deviation) than the IG mean value and standard deviation of the simulations with penalization terms (PT_#_). This happens because the big quantity of occupied cells close to the areas with higher belief makes many of the solutions within the PT_#_ approaches unfeasibly and reduces the global searching capabilities of CEO to the few feasible solutions sampled in the first iterations of CEO. Meanwhile, the sampling mechanism of the AS approach permits it to explore a bigger number and variety of feasible solutions in all the iterations of CEO and therefore, to obtain better solutions usually Figure 10e shows that the results of the simulations with the AS approach and with *M* = 2 are better (reach a higher IG mean value earlier) than the results of the simulation with *M* = 1. This happens because in the simulation with two agents, the system makes one stay collecting the left high probability region and moves the other over the left high probability region while going towards the right high probability region, while in the simulation with only one agent the system has to make it collect the first probability region completely before moving it to the second. Additionally, it is worth noting that both curves present a similar behavior: they interlace two increasing slopes with two plateaus. The first slope corresponds to the collection of the belief associated to the left high probability region, the first plateau to the displacements of the robots towards the right high probability and the second slope, to the collection of the right high probability region.

The graphics of Figure 11 show the trajectories of the robots (either over the belief or the map) and the updated belief at different time steps *k* of a representative simulation obtained with the AS approach. Each time step is represented in one row, while the first two columns are associated to the simulation with *M* = 1 robot and the last two columns to the simulation with *M* = 2 robots. The representation of the trajectories over the map shows that the robots are able to avoid the obstacles successfully. The representations of the trajectories over the updated belief show, in the simulations with one agent, how the robot almost collects the first probability mass before moving towards the second, while in the experiments with two agents, how one agent stays in the first high probability region while the other moves towards the second (after collecting initially some probability of the first probability mass).

#### 5.2.2. Dynamic Scenario

The characteristics and results of the dynamic scenario selected for this experimental setup are shown in Figures 12 and 13. According to the graphics represented in Figure 12a,b, the elements of the map are the walls of three corridors, and the initial object location belief defined over a target space Ω of 40 × 40 cells is concentrated in a single region over the middle of the left corridor. Again, the scenario is tested with different numbers of robots (*M* = 1 and *M* = 2), each starting in a position of one the two vertical corridors, and with the 4 restriction handling approaches (AS, PT_1_, PT_2_ and PT_3_) within ET-CEO. Finally, the target is moved, through the existing corridors, using a random transition model (whose probability table is generated once, before all the simulations are carried out, and only permits movements from each cell to its neighbors), and the simulations consist on 30 time steps, divided in 3 controller optimizations of horizon *N* = 10.

The curves presented in Figure 12c–e compare the IG value of the simulations with the 4 restriction handling approaches and with *M* = 1 and *M* = 2 agents. Figure 12c,d show that the results obtained by the four restriction handling approaches are similar, because the corridors are wide enough to generate in the RT_#_ approaches a smaller number of non-feasible solutions and the best solution found for the AS approach. Figure 12e shows that after *k* = 18, the results of the simulation with *M* = 2 are slightly better than the results of the simulation with *M* = 1. The improvement does not occur before, because the second agent, placed in the right corridor, is not able to reach earlier the region where there is some probability of finding the target. The improvement is small, because when it reaches that region, there is not much probability left by the first agent in the horizontal corridor. The maximum IG mean value reached within the simulation is below 0.65, because the random values of the transition probability model make the target probability spread over the corridors too quickly for the range and detection capability of the ultrasonic scanner. Finally, it is worth noting that the standard deviation of IG is negligible (smaller than 0.004) in all the cases due to the continuous drift of the belief that drives the robot to similar positions, from the ET point of view, at the end of each section of the trajectory.

The graphics of Figure 13 show the trajectories of the robots and the updated belief at different time steps *k* for a representative simulation obtained with the AS approach. Again, each time step is represented in one row, while the first two columns are associated to the simulation with *M* = 1 and the last two columns to the simulation with *M* = 2 robots. The representation of the trajectories over the map show that they are successfully confined by the MTS system to the corridors. The graphics of the trajectory over the updated belief for the single-agent simulation show how the robot collects the probability belief that is moving upwards through the left corridor, while a small probability mass is left in the horizontal corridor. The graphics of the trajectories over the updated belief for the multi-agent simulation show how one of the robots collects the probability belief that is moving upwards through the left corridor, while the other collects, after reaching the left end of the horizontal corridor, the small probability mass placed in that area.

### 5.3. Searching a Flat Object in an Orientation-Dependent Scenario with a Camera

This experimental setup is defined by a three dimensional grid, where each cell represents a cubic region associated to a range of the (*x*, *y*, *θ*) possible location and orientation of the agent. Again, we want to highlight that the orientation *θ* of the agent refers to the orientation of the vector normal to its surface. The search space is discretized using one meter increments in the (*x*, *y*) locations, and steps of 45 degrees in the orientation *θ*, which match the orientation increments of the agent orientations (associated to the eight cardinal directions) in order to permit the same granularity in the orientations of the target and the agent. With this discretization and the properties of the camera and vision identification system represented in Figure 4, the sensor, which has a distance coverage slightly higher than 10 m, is able to detect, with a certain probability, an object situated in the cells that fall inside the field of view equilateral triangular region (of 10 cells of height). The detection probability reaches its maximal value at a given distance, when the agent and target normal vector are parallel to each other, and has a zero value in the perpendicular case. Therefore, the scenarios analyzed in this experimental setup will let us determine if the MTS system is able to work in three dimensional searching spaces, with a complex sensor with a behavior dependent on the distance and relative orientation of the target and agent.

#### 5.3.1. Static Scenario

The characteristics and results of the static scenario selected for this experimental setup are shown in Figure 14. According to the graphics represented in Figure 14a,c,f, the initial target probability over a target space Ω of 40 × 40 × 8 cells is defined by two gaussian probability masses that are placed in two different locations of the space concentrated with two different disjoint orientations (North and East) to be able to analyze better the influence of the target orientation on the MTS approach. With this probability setup, we initially believe that the target is either placed (1) oriented towards the East at the upper center of the region under search or (2) oriented towards the North at its lower center. Again, the scenario is tested with different numbers of agents (*M* = 1 and *M* = 2), which are placed close to each of the probability mass regions, and the simulations consist on 30 time steps, divided in 3 controller optimizations of horizon *N* = 10.

The curves presented in Figure 14b compare the IG values of the simulations with *M* = 1 and *M* = 2 agents. The results of the simulation with *M* = 2 are much better (reaches a higher IG mean value)than the results of the simulation with *M* = 1. This happens because in the simulation with two agents, each of them becomes responsible of collecting the information of one probability mass, while in the simulation with a unique agent, the existing agent focuses the search only over the probability mass closer to it. The IG standard deviation, small but appreciable when zooming in at the intermediate time steps *k*, is associated to the heuristic and myopic behavior of the ET-CEO MTS system. Its narrow shape is due to the fact that in spite of the small differences of IG in the simulations the final positions of each segment of the trajectories allows the agent to gain a similar quantity of information in the future. Finally, it is worth noting that the IG curves of the simulations with *M* = 1 and *M* = 2 agents present a stepped behavior, of small increments followed by short plateaus. This short plateaus, which are by themselves a new behavior of the IG curves that has not been observed in the other experimental setups, are associated to those time steps of the simulation when the agents have a really little chance to detect the target because their relative (oblique) orientation prevents it.

The remaining graphics (Figure 14d,e,g,h) show the trajectories of the robots and updated belief in the two possible orientations of the target at time step *k* = 30 for two representative simulations. Each angular component is represented in one row and the results of the selected simulations with different number of agents are shown in consecutive columns. The graphics on the second column (single-agent simulation) confirm how the trajectory of the existing agent concentrates in the lower central probability mass, while the graphics on the third column (double-agent simulation) confirm how the trajectories of each agent concentrate in different probability masses. Additionally, it is interesting to observe the directions of the trajectories over each probability mass, and its relation with the target orientation associated to each of them. The red trajectory of the first agent that appears over the lower probability mass that is oriented towards the North has several vertical segments that are oriented towards one of the agent directions, where $\cos(\theta_{\tau }^{k}-\theta_{i}^{k})=1$ for $\theta_{\tau }^{k}=\text{North}$. The green trajectory of the second agent that appears over the upper probability mass which is oriented towards the East, has several horizontal segments, oriented again towards one of the agents directions where $\cos(\theta_{\tau }^{k}-\theta_{i}^{k})=1$ for $\theta_{\tau }^{k}=\text{East}$. The appropriated orientation of the robot over each probability mass can also be observed in the final belief: the probability mass of the upper center mass (oriented towards the East) is mainly eliminated using the field of view of a camera oriented towards the East and the West, while the probability mass of the lower center mass (oriented towards the North) is mainly eliminated using the field of view of a camera oriented towards the North and the South. The oblique orientation of some parts of the trajectory are used to move the agents efficiently (with at least some probality of detecting the target) to adjacent regions of the space. Therefore, the results show that not only does the MTS system send the agents to cover the probability regions, but also, it sends them to cover them in the most efficient way.

#### 5.3.2. Dynamic Scenario

The characteristics and results of the dynamic scenario selected for this experimental setup are shown in Figures 15, 16 and 17. The similarities that exist between the properties (initial target belief, initial agents location, number of agents, and simulation length) of this scenario and the previous can be observed in Figure 15a,c,d. The main difference is the existence of a transition model that moves each of the probability masses of the target belief over the location space associated to its corresponding orientation component. In other words, we use a different transition probability model for each orientation and do not allow the transition of the belief that exists in one orientation to another. Additionally, the transition probability model associated to the component oriented towards the North (placed in the lower center region) makes it move towards the South-West, while the transition probability model associated to the component oriented towards the East (placed in the upper center region) makes it move towards the North-East.

The curves presented in Figure 15b compare the IG mean values of the simulations with *M* = 1 and *M* = 2 agents. Again, the results of the simulation with *M* = 2 are much better than the results of the simulation with *M* = 1. The reason is similar: in the simulation with two agents, each agent becomes responsible of observing and following one probability mass, while in the simulation with a unique agent, the existing agent focuses the search only over the moving probability of the mass initially closer. The IG mean curves of the dynamic experiment are smoother than in the static case, due to the continuous dispersion of the probability masses, which lets the agents have more chances of detecting the target in the oblique displacements. Finally, the IG standard deviation is only slightly appreciable, when zooming in, at the intermediate time steps *k.* Again, this IG standard deviation is associated to the heuristic and myopic behavior of the ET-CEO MTS system.

Finally, the graphics in Figures 16 and 17 show the trajectories of the robots and updated belief in the two possible orientation of the target at different time steps *k* in a representative simulation for this scenario. The graphics within each figure are associated to the selected simulations with the same number of agents, each row corresponds to the same angular component of the belief space and each column is related to the same time step *k.* The distribution of the agent over the probability masses is similar to the distribution that occurs in the previous scenario. The trajectories are different, because the MTS system forces them to follow the probability masses and to orient towards the directions of maximum likelihood for each probability mass. The combination of both objectives, fulfilled by the MTS system through the definition of the ET utility function and this problem likelihood function, makes the agent following the lower probability mass (which is oriented towards the North and moves towards the South-West) move towards the South-West, alternating South-Western oblique displacements (to follow the probability mass) with vertical movements (to achieve the maximum likelihood benefit). Similarly, the agent following the upper probability mass (which is oriented towards the East and moved towards the North-East) moves towards the North-East alternating North-Eastern oblique displacements with horizontal movements. In the figures of these simulations it is also possible to see the shapes associated to the camera fields of view oriented towards the better direction for each probability mass.

### 5.4. Computation Cost

The computation costs of the controller of the ET-CEO system presented in this paper for the scenarios analyzed in the previous sections are summarized in the last column of Table 1. Its first two columns are used to identify each scenario, the third shows the number of cells associated to each region under search Ω, the forth the number of agents *M*, and the fifth the number of solutions sampled in each iteration of CEO. In all the scenarios the horizon is fixed to *N* = 10 time steps, the number of possible actions |Δ| = 8 and the iterations of CEO for each planning step is fixed to 20.

Before analyzing the results, we want to point out that the radar static scenario a special case within the computation cost comparison because its number of cells is different to the number of cells of its dynamic counterpart. Besides, the maximum number of iterations does not need to coincide, as it happens in this paper, with the number of simulations used to statistically analyze the performance of the ET-CEO system. Finally, for comparison purposes the computation time in the table is always associated to the maximum number of iterations, although during the experiments less iterations have been needed sometimes.

The computation times are obtained, as well as the results presented in the previous sections, with a Matlab implementation of the ET-CEO system running in a Parallels Windows XP virtual machine with 6 cores of a 2.4 GHz Intel Core i7 Macbook Pro. In order to speed up the computation of the ET function presented in Algorithm 2 we pre-calculate some information associated to the probabilistic sensorial models and carry out the summation Σ_*τ*^*k*+*j*^ϵΩ_*P*(*τ^k^*^+^*^j^*^+1^|*τ^k^*^+^*^j^*)*b̂*(*τ^k^*^+^*^j^*) only over the states with *P*(*τ^k^*^+^*^j^*^+1^|*τ^k^*^+^*^j^*) ≠ 0. Besides, we carry out the evaluation of ET over the E samples of each CEO iteration in parallel over the 6 cores of the Windows XP virtual machine using the Matlab Parallel Computing Toolbox.

The results of the table show the following patterns:

- Within the same scenario, the number of samples/solutions *E* to be evaluated by ET-CEO in each iteration is proporcional to the number of agents *M.* Moreover, although the number of solutions to be evaluated in the scenarios with *M* = 4 doubles the number of solutions with *M* = 2 and the number of solutions with *M* = 2 doubles the number of solutions with *M* = 1, in each scenario (sensor type+static/dynamic) the relationship among computation time used by the 20 generations of the ET-CEO is higher than the double (for instance in the radar static case, the computation cost is 93, 29 and 13 s, and the relation between these numbers are 3.2 and 2.23). This happens because each solution (set of agent actions) has more values to be sampled and it is harder to evaluate because as the number of agents grow the number of sensorial models to be applied in the 4th step of Algorithm 2 is incremented.
- The increment in the computation time that occurs as the number of agents grows within the same scenario is different for each type of sensors. To compute it, we take into account the duplication (*2) of the number of ET evaluation (for instance in the radar dynamic case the increment between *M* = 2 and *M* = 1 is 35 − 15 × 2 = 5 s while in ultrasonic scanner static case is 62 − 20 × 2 = 12 s). This increment is associated to the differences in the computation time of each sensor model: we have been able to pre-calculate almost all the operations associated to the radar, but have to continuously recalculate the part of the sonar associated with the map and the part of the camera associated with its field of view.
- For the same sensor type and for the same number of agents *M*, the dynamic experiments requires higher computation time than its static counterpart (for instance, the sonar static experiment requires 20 s while the sonar dynamic experiment requires 30). This extra time is associated to the prediction/transition 6th step of Algorithm 2 that can be substituted by *f*(*τ*^*k*+*j*+1^) = *b̂*(*τ*^*k*+*j*^) in the static scenarios because in those cases *P*(*τ^k^*^+^*^j^*^+1^|*τ^k^*^+^*^j^*) = *δ*(*τ^k^*^+^*^j^*^+1^ *− τ^k^*^+^*^j^*).

In short, increasing the number of agents, augmenting the complexity of the sensorial model and solving dynamic scenarios causes an increment in the computation cost of the ET-CEO system. Nevertheless, the times used to compute the solutions are lower than 5 min in the hardest problem setup (camera+dynamic) in a computer with 6 cores. These times can be improved further by implementing the code in C (which can reduce the computation time one order of magnitude) and by parallelizing the operations of the ET evaluation (Algorithm 2) using more cores or a Graphics Processing Unit (GPU) to achieve real-time computation [56,57].

### 5.5. Overall Discussion

The previous simulations show that the ET-CEO based autonomous and intelligent system presented in the paper is able to guide the agents towards the regions of the search space where there is a positive probability of finding the target. Moreover, the trajectories described by the agents in based of the control signals obtained by the controller layer seem appropriated for the provided sensor models and scenarios. For instance, in the static case of the radar experimental setup (see Figure 6) the UAVs are controlled accordingly with a covering strategy of the areas where the probability mass is concentrated. Moreover, the trajectories of the UAVs using the ideal sensor are packed more tightly than those followed by the UAVs with the complex radar. Both behaviors are expected in this scenario: the covering strategy minimizes the searching time preventing the trajectories to pass by already sensed regions, while the shorter distance range of the ideal sensor makes its covering tighter. In the radar dynamic case (see Figures 8 and 9) the UAVs follow the movements of the probability mass and distribute themselves to capture the probability from different points. Again, the behavior that emerges from the controller action is reasonable for the scenario. In the simulations with the ultrasonic scanner we observe how the agents are displaced according to a trajectory that lets them collect the probability masses efficiently. Moreover, in the static example (see Figure 11) the controller is able to identify a trajectory between the obstacles that lets the robots collect the first probability mass while moving towards the second, while in the dynamic case (see Figure 13), one robot follows the probability mass moving through the left corridor while the other intercepts the probability mass moving across the horizontal one. Again, the behavior is reasonable to minimize the expected time to detect the target in both scenarios. Finally, the trajectories obtained in the simulations with the camera and vision identification system orient the agent and camera towards the orientation that makes the target more visible. In the static case (see Figure 14) this is achieved by moving the agents with a pattern that lets them cover the region of the probability mass while observing the object as much as possible. In the dynamic case (see Figures 16 and 17) the trajectories follow the displacement of the mass and re-orients the agents to let them observe the object as much as possible. Again, the emergent behavior is a good solution to optimize the expected detection time.

However, the result obtained by the ET+CEO controller are not always the optimal, as the variability (standard deviation) observed in the IG analysis show. As we have already pointed out, the suboptimality of the solutions is associated, mainly, to the following reasons. On one hand, the inherent suboptimality behavior of CEO, which has a high capability of finding good solutions, but that does not necessary returns the best. On the other, the evaluation of ET over a fixed horizon of *N* = 10 time steps, only takes into account the subspace of target states reachable from the current agent locations with *N* actions. Therefore, the complete trajectory originated by our controller is a concatenation of myopic solutions.

The simulations show two interesting behaviors. On one hand, the selected static scenarios show a higher IG standard deviation than their dynamic counterparts. Although the origin of this difference can be partially associated to the original belief and agents positions of each scenario, we believe that the displacement of the probability caused by the dynamics of the target make the agents follow the direction of the probability drift and reduce the IG variability. On the other, the simulations of the sonar show that some scenarios with restricted control signals are better tackled with a CEO with an adapted sampling mechanism than with a CEO with a penalization of the evaluation function.

The computation time associated to the scenarios under test varies due to the number *M* and types of sensors and the static/dynamic behavior of the target. It is also dependent on the number of cells used to divide the space under search Ω and on the horizon time steps *N.* A careful implementation of the ET-CEO controller, which evaluates in parallel the ET of subsets of solutions sampled in each iteration of CEO, is capable of obtaining a solutions every 99, 74 and 257 s for the more demanding scenarios (dynamic with the highest number of agents) or the radar, sonar and camera experimental setups. If necessary, these times could be further reduced by a higher parallelization of the ET evaluation. Alternatively, the computation time can also be reduced decreasing the horizon value *N*, at the expense of incrementing the myopic behavior of the solutions. Another common way of dealing with the myopia of solutions in receding controllers consists on re-calculating the action plan after executing only one action. This strategy could be followed in our approach too as long as the computation time required to calculate each plan is shorter than the execution of each action.

Finally, based on the overall behavior of the trajectories and in spite of the inherent suboptimality and myopia of the solutions, we believe that the ET-CEO based approach is a good generic solution for complex observation models.

## 6. Conclusions

The paper studies the applicability of the autonomous and intelligent system consistent of a data fusion layer driven by a RBF and a controller layer implemented over a CEO that minimizes the detection ET to MTS problems with complex sensorial detection probability models.

The testing problems consists on searching a target with multiple agents using either (1) a radar working in a space free of obstacles; (2) an ultrasonic scanner in a space with known static obstacles; or (3) a camera and vision identification system whose sensing capabilities take into account the relative orientation of the target with respect the camera. Each sensor has a sensorial detection model with different properties, ranging from stepped detection probabilities to continuous/discontinuous differentiable/non-differentiable detection probabilities dependent always on the distance between the target and the robot, and in some cases on the relative orientation of the target with respect the agent or on the presence of occluding objects. Therefore, the selection permits us to test the ET-CEO approach using a big variety of sensorial behaviors that appear in real world searching tasks. The statistical analysis of the results, obtained under simulation for different scenarios for each sensor, show some variability in the solutions, which is associated to the inherent suboptimality of CEO and to the myopia of the ET evaluation. The representative simulations show how the ET-CEO based algorithm, designed as a general controller that exploits only the probability models of the problem, distributes efficiently the agents over the searching region to detect the targets.

There are several future lines of research to be followed after having validated the applicability of the ET-CEO approach in complex sensorial-modeled searching tasks that are defined over discrete finite action and search space. The most direct consists on analyzing the performance of the approach with multiple types of sensors simultaneously and with the data simulated by the sensors (instead of assuming non-detection during the simulations). Another option could be extending the approach to continuous action space models, substituting the optimization algorithm (CEO) for an equivalent version for the continuous space (selected, for instance, from the estimation of distribution algorithm family [58]). The search space could be made also continuous, selecting an appropriated RBF. Alternative, we can try to include in the ET definition a generic heuristic term, capable of reducing the myopia associated to finite-horizon controllers, following the lines presented in [11,59]. Finally, the future improvements of the system should be validated with complex sensorial models as the ones presented in this paper, to be able to asses, if the generality of the system remains unaffected.

## Figures and Tables

**Table 1.** Computations cost of the controller for the scenarios (with *N* = 10 and |Δ| = 8).

**Scenario**		#*_cells_*	*M*	*E* = 10 × *M* × *N* × |Δ|	**Computation Time (seg)**
Radar	Static	80 × 80	1	800	13
			2	1600	29
			4	3200	93

	Dynamic	40 × 40	1	800	15
			2	1600	35
			4	3200	99

Sonar	Static	40 × 40	1	800	20
			2	1600	62

	Dynamic	40 × 40	1	800	30
			2	1600	74

Camera	Static	40 × 40 × 8	1	800	60
			2	1600	219

	Dynamic	40 × 40 × 8	1	800	80
			2	1600	257

## Appendix

This Appendix shows the derivation of the data fusion layer, controller layer and information gain expressions. To obtain them, we exploit the probability properties presented in Table A1, where each row holds a different property, the first column describes it shortly, the second column stores the property identifier used hereafter, and the third column holds the mathematical expression of the operation. Within the expressions, *a, b, c* and *x^i^* stand for generic variables.

**Table A1.** Basic probability operations.

**Description**	**Identifier**	**Expresion**
Bayes Rule	BR	*P*(*a,b|c*) = *P*(*a|b, c*)*P*(*a|c*)
Marginalization Rule	MR	$P(a|b)=\sum_{c}P(a,c|b)$
Complement Rule + De Morgan's Law	∪ → ∩	$P(\underset{i=1:N}{\cup }x^{i})=1-P({\bar{x}}^{1:N})$

### A1. Data Fusion Layer Expressions

The expressions of the prediction and update steps alternated in the data fusion layer are obtained following the usual derivation of the RBF [9,10].

#### A1.1. Prediction Step

The prediction operation, presented in Equation (1), is derived in the following expressions, where Equation (A1) incorporates the variable *τ^k^*^−1^ using the MR property, Equation (A2) applies BR to separate the previous time step belief $P(\tau^{k-1}|z_{1:M}^{1:k-1},s_{1:M}^{0:k},\in )$ from the rest of the expression, and Equation (A3) exploits the probability independence of *τ_k_* on the variables z1:M1:k−1 and $s_{1:M}^{0:k}$ given *τ_k_*_−1_ to substitute $P(\tau^{k}|\tau^{k-1},z_{1:M}^{1:k-1},s_{1:M}^{0:k},\in )$ by the transition model *P*(*τ^k^*|*τ^k^*^−1^, *ϵ*) and the fact that $b(\tau^{k-1})≜P(\tau^{k-1}|z_{1:M}^{1:k-1},s_{1:M}^{0:k},\in )$.

(A1) $$P(\tau^{k}|z_{1:M}^{1:k-1},s_{1:M}^{0:k},\epsilon )=\sum_{\tau^{k-1}}P(\tau^{k},\tau^{k-1}|z_{1:M}^{1:k-1},s_{1:M}^{0:k},\epsilon )$$

(A2) $$=\sum_{\tau_{k-1}\in \Omega }P(\tau^{k}|\tau^{k-1},z_{1:M}^{1:k-1},s_{1:M}^{0:k},\epsilon )P(\tau^{k-1}|z_{1:M}^{1:k-1},s_{1:M}^{0:k},\epsilon )$$

(A3) $$=\sum_{\tau_{k-1}\in \Omega }P(\tau^{k}|\tau^{k-1},\epsilon )b(\tau^{k-1})$$

#### A1.2. Update Step

The update operation, presented in Equation (2), is obtained in the following expressions, where Equation (A4) holds the probability definition of the target belief *b*(*τ^k^*) at time step *k*, Equation (A5) applies BR to move the variables $z_{1:M}^{k}$ to the left side of the probability in the numerator, and Equation (A6) uses MR to include the variable *τ^k^* in the left side of the probability of the denominator. The remaining operations are carried out in the numerator and within the summation term of the denominator: Equation (A7) applies BR to separate the predicted belief $P(\tau^{k}|z_{1:M}^{1:k-1},s_{1:M}^{0:k},\in )$ from the rest, and the Equation (A8) exploits the fact that the measurements $z_{i}^{k}$ are independent on each other given the agent $s_{i}^{k}$ and target *τ^k^*.

(A4) $$b(\tau^{k})≜P(\tau^{k}|z_{1:M}^{1:k},s_{1:M}^{0:k},\epsilon )$$

(A5) $$=\frac{P(\tau^{k},z_{1:M}^{k}|z_{1:M}^{1:k-1},s_{1:M}^{0:k},\epsilon )}{P(z_{1:M}^{k}|z_{1:M}^{1:k-1},s_{1:M}^{0:k},\epsilon )}$$

(A6) $$=\frac{P(\tau^{k},z_{1:M}^{k}|z_{1:M}^{1:k-1},s_{1:M}^{0:k},\epsilon )}{\Sigma_{\tau^{k}\in \Omega }P(\tau^{k},z_{1:M}^{k}|z_{1:M}^{1:k-1},s_{1:M}^{0:k},\epsilon )}$$

(A7) $$=\frac{P(z_{1:M}^{k}|\tau^{k},z_{1:M}^{1:k-1},s_{1:M}^{0:k},\epsilon )P(\tau^{k}|z_{1:M}^{1:k-1},s_{1:M}^{0:k},\epsilon )}{\sum_{\tau^{k}\in \Omega }P(z_{1:M}^{k}|\tau^{k},z_{1:M}^{1:k-1},s_{1:M}^{0:k},\epsilon )P(\tau^{k}|z_{1:M}^{1:k-1},s_{1:M}^{0:k},\epsilon )}$$

(A8) $$=\frac{\Pi_{i=1:M}P(z_{i}^{k}|\tau^{k},s_{i}^{k},\epsilon )P(\tau^{k}|z_{1:M}^{1:k-1},s_{1:M}^{0:k},\epsilon )}{\sum_{\tau^{k}\in \Omega }\Pi_{i=1:M}P(z_{i}^{k}|\tau^{k},s_{i}^{k},\epsilon )P(\tau^{k}|z_{1:M}^{1:k-1},s_{1:M}^{0:k},\epsilon )}$$

### A2. Control Layer Expressions

The expressions of the functions required to calculate the truncated expected time utility function optimized by the controller layer are obtained following the same strategy that we present in [13]. In this appendix, their derivation is presented with higher detail.

#### A2.1. Expected Time

According to [60], the Expected Time (ET) to detect the target can be calculated with Equation (A9), where *P*(*k* ≤ *t* ≤ *k* + *j*)) represents the probability of detecting the target at any time step between *k* and *k* + *j*. This probability can be calculated as $P(\cup_{i=1:M,l=1:j}D_{i}^{k+l}|s_{1:M}^{1:k+j},z_{1:M}^{1:k},\epsilon )$, *i.e.*, as the probability of detecting the target with any of the agents at any time step between *k* and *k* + *j*. Next, Equation (A11) is obtained using the ∪ → ∩ property and transformed into Equation (A12) using MR to include *τ_k_*_+_*_j_*. The following step, shown in Equation (A13), exploits BR and assumes that the no-detection values of the measurements at time step *k* + *j* only depend on the target and agent state at the same time step. Equation (A14) is obtained taking into account the independence of the measurements $z_{i}^{k}$ on each other given the agent $s_{i}^{k}$ and target *τ^k^*, and considering that the joint probability of the no-detection measurements from *k* + 1 to *k* + j − 1 and of the target state at *k* + *j* is independent on the agent state at *k* + *j*. Finally, in Equation (A15) we only redefine $P({\bar{D}}_{1:M}^{k+1:k+j-1},\tau^{k+j}|s_{1:M}^{1:k+j-1},z_{1:M}^{1:k},\epsilon )$ as *f*(*τ^k^*^+^*^j^*).

(A9) $$\text{ET}(s_{1:M}^{k+1:k+\infty })=\sum_{j=1}^{\infty }\left(1-P(k\leq t\leq k+j)\right)$$

(A10) $$=\sum_{j=1}^{\infty }\left(1-P\left(\underset{i=1:M,l=1:j}{\cup }D_{i}^{k+l}|s_{1:M}^{1:k+j},z_{1:M}^{1:k},\epsilon \right)\right)$$

(A11) $$=\sum_{j=1}^{\infty }P\left({\bar{D}}_{1:M}^{k+1:k+j}|s_{1:M}^{1:k+j},z_{1:M}^{1:k},\epsilon \right)$$

(A12) $$=\sum_{j=1}^{\infty }\sum_{\tau^{k+j}\in \Omega }P\left({\bar{D}}_{1:M}^{k+1:k+j},\tau^{k+j}|s_{1:M}^{1:k+j},z_{1:M}^{1:k},\epsilon \right)$$

(A13) $$=\sum_{j=1}^{\infty }\sum_{\tau^{k+j}\in \Omega }P\left({\bar{D}}_{1:M}^{k+j}|\tau^{k+j},s_{1:M}^{k+j},\epsilon \right)P\left({\bar{D}}_{1:M}^{k+1:k+j-1},\tau^{k+j}|s_{1:M}^{1:k+j},z_{1:M}^{1:k},\epsilon \right)$$

(A14) $$=\sum_{j=1}^{\infty }\sum_{\tau^{k+j}\in \Omega }\prod_{i=1}^{M}P\left({\bar{D}}_{i}^{k+j}|\tau^{k+j},s_{i}^{k+j},\epsilon \right)P\left({\bar{D}}_{1:M}^{k+1:k+j-1},\tau^{k+j}|s_{1:M}^{1:k+j-1},z_{1:M}^{1:k},\epsilon \right)$$

(A15) $$=\sum_{j=1}^{\infty }\sum_{\tau^{k+j}\in \Omega }\prod_{i=1}^{M}P\left({\bar{D}}_{i}^{k+j}|\tau^{k+j},s_{i}^{k+j},\epsilon \right)f\left(\tau^{k+j}\right)$$

The infinite terms of the expression obtained for computing ET make it intractable. However, as Equation (A9) is valid for probability functions that sum less than one [61], we can use it to calculate ET for a limited horizon of *N* time steps. In order to do that, we only need to substitute ∞ by *N* in the external summation of Equations (A9)–(A15).

#### A2.2. Joint Target and No Detection Probability

The expression obtained for the ET is a function of *f*(*τ^k^*^+^*^j^*), that represents joint probability of the target at time step *k* + *j* and the no-detection measurements from *k* + 1 up to *k* + *j* − 1. The recursive Equations (5) and (6) used to calculate *f*(*τ^k^*^+^*^j^*) are derived next.

- The general case is derived through Equations (A16)–(A20). First, Equation (A16) shows the definition of *f*(*τ^k^*^+^*^j^*) and Equation (A17) uses MR to include the variable *τ^k^*^+^*^j^*^−1^ in the probability function. Next, Equation (A18) exploits BR to separate the transition model from the rest and Equation (A19) applies again BR to separate the probability of the no-detection measurements at *k* + *j* − 1 from the rest. Finally, Equation (A20) assumes that the no-detection measurements at *k* + *j* are independent on each other given given the agent $s_{i}^{k}$ and target *τ_k_*, considers that the joint probability of the no-detection measurements from *k*+1 to *k*+*j* − 2 and of the target state at *k*+*j* − 1 is independent on the agent state at *k* + *j* − 1, and redefines $P(\tau^{k+j-1},{\bar{D}}_{1:M}^{k+1:k+j-2}|s_{1:M}^{1:k+j-2},z_{1:M}^{1:k})$ as *f*(*τ^k^*^+^*^j−^*^1^).
  (A16) $$f(\tau^{k+j})=P\left(\tau^{k+j},{\bar{D}}_{1:M}^{k+1:k+j-1}|s_{1:M}^{1:k+j-1},z_{1:M}^{1:k},\epsilon \right)$$
  (A17) $$=\sum_{\tau^{k+j-1}\in \Omega }P\left(\tau^{k+j},\tau^{k+j-1},{\bar{D}}_{1:M}^{k+1:k+j-1}|s_{1:M}^{1:k+j-1},z_{1:M}^{1:k},\epsilon \right)$$
  (A18) $$=\sum_{\tau^{k+j-1}\in \Omega }P\left(\tau^{k+j}|\tau^{k+j-1}\right)P\left(\tau^{k+j-1},{\bar{D}}_{1:M}^{k+1:k+j-1}|s_{1:M}^{1:k+j-1},z_{1:M}^{1:k}\right)$$
  (A19) $$=\sum_{\tau^{k+j-1}\in \Omega }P\left(\tau^{k+j}|\tau^{k+j-1}\right)P\left({\bar{D}}_{1:M}^{k+j-1}|\tau^{k+j-1},s_{1:M}^{k+j-1}\right)P\left(\tau^{k+j-1},{\bar{D}}_{1:M}^{k+1:k+j-2}|s_{1:M}^{1:k+j-1},z_{1:M}^{1:k}\right)$$
  (A20) $$=\sum_{\tau^{k+j-1}\in \Omega }P\left(\tau^{k+j}|\tau^{k+j-1}\right)\prod_{i=1}^{M}P\left({\bar{D}}_{i}^{k+j-1}|\tau^{k+j-1},s_{i}^{k+j-1}\right)f\left(\tau^{k+j-1}\right)$$
- The basic case, which occurs when *j* = 1, is derived through Equations (A21)–(A25): Equation (A21) shows the particularization of the definition of *f*(*τ^k^*^+^*^j^*) for *j* = 1. The lack of measurements in the range from *k* + 1 up to *k* is stated in Equation (A22). Equation (A23) is obtained using MR to include the variable *τ_k_* in the probability function and Equation (A24) is derived exploiting BR to separate the transition model from the probability of the target state at time step k, given the past measurements and agents trajectory This last probability equals the target belief at time step *k*, as Equation (A25) shows.
  (A21) $$f(\tau^{k+1})≜P\left(\tau^{k+1},{\bar{D}}_{1:M}^{k+1:k}|s_{1:M}^{1:k},z_{1:M}^{1:k},\epsilon \right)$$
  (A22) $$=P\left(\tau^{k+1}|s_{1:M}^{1:k},z_{1:M}^{1:k},\epsilon \right)$$
  (A23) $$=\sum_{\tau^{k}\in \Omega }P\left(\tau^{k+1},\tau^{k}|s_{1:M}^{1:k},z_{1:M}^{1:k},\epsilon \right)$$
  (A24) $$=\sum_{\tau^{k}\in \Omega }P\left(\tau^{k+1}|\tau^{k},\epsilon \right)P\left(\tau^{k}|s_{1:M}^{1:k},z_{1:M}^{1:k},\epsilon \right)$$
  (A25) $$=\sum_{\tau^{k}\in \Omega }P\left(\tau^{k+1}|\tau^{k},\epsilon \right)b\left(\tau^{k}\right)$$

### A3. Information Gain Expression

The IG is derived in next expressions, where Equation (A26) shows the formal definition of the IG, Equation (A28) exploits the ∪ → ∩ property and Equation (A28) assumes the same steps as the inner summation of Equation (A11), that $f(\tau^{k})=\sum_{\tau^{j-1}\in \Omega }P(\tau^{j}|\tau^{k-1})\prod_{i=1}^{M}P({\bar{D}}_{i}^{k-1}|\tau^{k-1},s_{i}^{k-1})$, *f*(*τ^k^*^−1^) and that *f*(*τ*^1^) = Σ_τ^0^∈Ω_ *P*(*τ*^1^|*τ*^0^, *ϵ*)b(*τ*^0^)).

(A26) $$IG(k)≜P\left(\underset{i=1:M,l=1:k}{\cup }D_{i}^{l}|s_{1:M}^{1:k},z_{1:M}^{1:k},\epsilon )\right)$$

(A27) $$=1-P\left({\bar{D}}_{1:M}^{1:k}|s_{1:M}^{1:k},\epsilon \right)$$

(A28) $$=\left[1-\sum_{\tau^{k}\in \Omega }\prod_{i=1}^{M}P\left({\bar{D}}_{i}^{k}|\tau^{k},s_{i}^{k},\epsilon \right)f\left(\tau^{k}\right)\right]$$
